# Invasive wild deer exhibit environmental niche shifts in Australia: Where to from here?

**DOI:** 10.1002/ece3.10251

**Published:** 2023-07-03

**Authors:** Catherine L. Kelly, Iain J. Gordon, Lin Schwarzkopf, Anna Pintor, Anthony Pople, Ben T. Hirsch

**Affiliations:** ^1^ College of Science and Engineering James Cook University Townsville Queensland Australia; ^2^ Fenner School of Environment & Society Australian National University Canberra Australian Capital Territory Australia; ^3^ James Hutton Institute Aberdeen UK; ^4^ CSIRO Australian Tropical Science and Innovation Precinct Townsville Queensland Australia; ^5^ Central Queensland University Townsville Queensland Australia; ^6^ Department of Agriculture and Fisheries Brisbane Queensland Australia; ^7^ Smithsonian Tropical Research Institute Panama Panama

**Keywords:** Cervidae, future spread, invasive species, niche shifts, species distribution modeling

## Abstract

Invasive species have established populations around the world and, in the process, characteristics of their realized environmental niches have changed. Because of their popularity as a source of game, deer have been introduced to, and become invasive in, many different environments around the world. As such, deer should provide a good model system in which to test environmental niche shifts. Using the current distributions of the six deer species present in Australia, we quantified shifts in their environmental niches that occurred since introduction; we determined the differences in suitable habitat between their international (native and invaded) and their Australian ranges. Given knowledge of their Australian habitat use, we then modeled the present distribution of deer in Australia to assess habitat suitability, in an attempt to predict future deer distributions. We show that the Australian niches of hog (*Axis porcinus*), fallow (*Dama dama*), red (*Cervus elaphus*), rusa (*C. timorensis*), and sambar deer (*C. unicolor*), but not chital deer (*A. axis*), were different to their international ranges. When we quantified the potential range of these six species in Australia, chital, hog, and rusa deer had the largest areas of suitable habitat outside their presently occupied habitat. The other three species had already expanded outside the ranges that we predicted as suitable. Here, we demonstrate that deer have undergone significant environmental niche shifts following introduction into Australia, and these shifts are important for predicting the future spread of these invasive species. It is important to note that current Australian and international environmental niches did not necessarily predict range expansions, thus wildlife managers should treat these analyses as conservative estimates.

## Graphical abstract

Deer have been introduced to, and become invasive in, many different environments around the world. We determined the differences in suitable habitat between their international (native and invaded) and their Australian ranges and modeled the present distribution of deer in Australia to assess habitat suitability to attempt to predict future deer distributions.

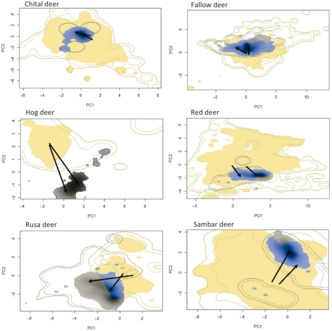

## 1 INTRODUCTION

Invasive species are one of the leading causes of ecological change and biodiversity loss worldwide (Doherty et al., 2016; Mack et al., 2000). Humans have facilitated the invasion, and subsequent spread, of non‐native species to previously inaccessible areas and niches, and these species have then gone on to become invasive (Da Re et al., 2020; Hernandez & Parker, 2018; Tingley et al., 2014). Here, a niche is defined as the range of ecological conditions in which a species can maintain viable populations (Guisan et al., 2014; Srivastava et al., 2020; Valverde et al., 2011). Factors such as propagule pressure can influence the probability of an invasive species' successful establishment in a novel environment (Prins & Gordon, 2014), while other mechanisms such as adaptation can allow a species to expand its environmental niche (Kolar & Lodge, 2001; Kumar et al., 2015; Lakeman‐Fraser & Ewers, 2013; Simberloff, 2009; Tingley et al., 2014). Understanding the degree to which species' environmental niches can change post‐introduction provides insights into invasion processes and assists with predicting areas vulnerable to future spread (Braschler et al., 2019; Guisan et al., 2014; Jourdan et al., 2021; Peterson, 2011).

As niche characteristics are often different between native and invaded environments, a species' ability to rapidly adapt to a novel environment can increase their probability of invasion and their likelihood of successful colonization (Gallagher et al., 2010; Guisan et al., 2014; Peterson & Nakazama, 2008; Sakai et al., 2001). Many species have expanded into environmental conditions that are not present in their native range, which could occur due to using a greater portion of their fundamental niche, exploiting phenotypic plasticity, or adapting to new conditions and spreading, defined here as undergoing an environmental niche shift (Beaumont et al., 2009; Blackburn & Duncan, 2001; Guisan et al., 2014; Jourdan et al., 2021; Pearman et al., 2008). Understanding the niche shifts that have occurred in species between their native and invasive distributions may help with understanding further range expansion and invasion potential (Broennimann & Guisan, 2008; Gonzalez‐Moreno et al., 2015).

Species distribution models (SDMs) are a standard method to predict habitat suitability and invasion risk (Santamarina et al., 2019; Tingley et al., 2014; Valverde et al., 2011). To fully understand invasion risk, it is important to model habitat suitability using a species' global invaded distribution. Because organisms may not use their entire potential (i.e., fundamental) niche, predicting suitable habitats based only on the native distribution can severely underestimate the potential for an invasive species' establishment or spread in a novel environment (Ahmad et al., 2019; Morehouse & Tobler, 2013; Srivastava et al., 2020; Tingley et al., 2014). When quantifying suitable habitat using SDMs, it is also important to consider habitat connectivity because suitable habitat may occur in areas nowhere near a species' site of introduction (Elith & Leathwick, 2009), making it difficult or impossible for the species to expand into them. For this reason, it is important to model habitat connectivity in conjunction with suitability (Dunstan & Johnson, 2007; Soberón & Peterson, 2005; Valverde et al., 2011). Here, we used SDMs and connectivity metrics to predict the invasiveness of a widespread group of introduced fauna.

Deer (order Artiodactyla) are a highly adaptable and diverse family that occupies various ecosystems around the world (Fautley et al., 2012; Fraser, 1996; Hudson & Jeon, 2003). Invasive deer can have severe impacts by degrading habitats, competing with native species, and spreading diseases and parasites (Dolman & Waber, 2008; Doran & Laffan, 2005; Ens et al., 2016; Hess, 2016). Invasive deer often have significant economic impacts, posing risks to motor vehicles, and competing with livestock for feed (Jesser, 2005; Kusta et al., 2017; McLeod, 2009). Despite this, humans have successfully established populations of deer globally (King, 2005), largely for game. Many deer now have broad international distributions (Forsyth & Hickling, 1998) and, like other species introduced to novel environments, can adapt, spread, and become invasive. Because deer have been so widely introduced internationally, and so often become invasive (Forsyth & Duncan, 2001; Hall & Hill, 2005; Long et al., 2003), it is important to calculate their niche using worldwide occurrences, as they are likely to occupy a large proportion of their fundamental niche space (i.e., realized niche). Here, we modeled deer distributions in Australia using their worldwide environmental niches to predict likely suitable habitat in Australia.

Deer were introduced to Australia in the early 1800s by acclimatization societies for hunting and to make the landscape more familiar for colonists (Bentley, 1967; Roff, 1960). Of the 29 species of deer brought to Australia (Table 1), six have established free‐living populations and increased in population size and range (Bentley, 1967; Moriarty, 2004). These species have successfully established in multiple ecosystems across Australia, and many deer species are negatively impacting local environments and economies (Burgin et al., 2014; Davis et al., 2016; English, 2007; Forsyth et al., 2012; Jesser, 2005). Predicting their future ranges is thus important for control and management.

**TABLE 1:** The 29 species (and subspecies) of deer brought to Australia (the six with wild distributions are in bold), and the states where they occurred.

Species	Latin name	First record	IUCN	States held in
Barasingha deer	*Cervus duvaucelli*	1864	V	VIC, NT
Bawean deer	*Axis kuhlii*	1867	CE	VIC
Chinese water deer	*Hydropotes inermis*	1867	V	VIC, SA
**Chital deer**	***Axis axis***	**1806**	**LC**	**VIC SA WA NSW QLD**
Eld's deer (Panolia deer)	*Cervus eldii*	1900	E	VIC
**Fallow deer**	***Dama dama***	**1832**	**LC**	**VIC SA WA NSW QLD NT**
**Hog deer**	***Axis porcinus***	**1860**	**E**	**VIC SA WA NSW**
Indian muntjac	*Muntiacus muntjak*	1863	LC	VIC, SA, WA
Tennasserim muntjac	*Muntiacus feae*	1926	DD	VIC
Mouse deer	*Moschiola meminna*	1878	LC	VIC, SA, QLD
Java mouse‐deer	*Tragulus jaranicus*	1864	DD	SA, NSW
Mule deer	*Odocoileus hemionus*	1863	LC	VIC
Black‐tailed deer	*Odocoileus hemionus columbianus*	1914	LC	VIC
Musk deer	*Moschus moschiferus*	1871	V	VIC
Pere David's deer	*Elaphurus davidianus*	1903	EX	WA, NSW
**Red deer**	***Cervus elaphus***	**1865**	**LC**	**VIC SA WA QLD NSW**
Reindeer	*Rangifer tarandus*	1891	V	VIC
Roe deer	*Capreolus capreolus*	1874	LC	VIC
**Rusa deer**	***Cervus timorensis***	**1865**	**V**	**VIC SA WA NSW QLD NT**
Batavia deer (Javan rusa)	*Cervus timorensis russa*	1868	V	VIC
Molucca deer	*Cervus timorensis moluccensis*	1891	V	VIC
**Sambar deer**	***Cervus unicolor***	**1860**	**V**	**VIC NSW NT**
Malay sambar	*Cervus unicolor equinus*	1898	V	VIC
Borneo deer	*Cervus unicolor brookei*	1883	V	VIC, SA
Sika deer	*Cervus nippon*	1868	LC	VIC
Formosa sika	*Cervus nippon taiouanus*	1863	LC	VIC
Visayan spotted deer	*Rusa alfredi*	1902	E	WA
Wapiti	*Cervus canadensis*	1886	LC	VIC, SA, WA, NSW
White‐tailed deer	*Odocoileus virginianus*	1877	LC	SA

*Note*: IUCN represents the IUCN Red List status as of publication.

Abbreviations: CE, critically endangered; DD, data deficient; E, endangered; EX, extinct in wild; LC, least concern; V, vulnerable.

To identify areas vulnerable to future invasion by deer in Australia, we created SDMs for the native and international ranges of the six established deer species. To examine if deer changed their environmental niches (i.e., had a niche shift) following their introduction into Australia, we quantified the extent of niche overlap between each species' international and Australian ranges. We predicted that species with broader international invasive distributions would exhibit fewer differences between their international range and their Australian distribution compared to those species with limited global distributions. We expected that species whose native range was most similar to available Australian habitats would have the largest potential for spread. Species that have exhibited niche shifts since being introduced to Australia may spread beyond habitat presently deemed suitable by our SDMs. While all species may have the capacity for further spread, those that have already had niche shifts may be less predictable in terms of their potential future distributions.

## 2 METHODS

### 2.1 Species records and environmental data

Species presence data for the six deer species that have established in Australia were obtained from open‐access databases. Native range and international occurrence records were collected from the Global Biodiversity Information Facility (GBIF; “GBIF.org”), and Australian occurrence records were collected from the Atlas of Living Australia (ALA; “ala.org.au”). We supplemented Australian records with direct observations from “FeralScan”, a citizen science platform to track feral deer observation records in Australia (“www.feralscan.org.au”). We also supplemented Australian records of chital deer (*Axis axis*) with occurrence records collected from 2017 to 2020 using direct observations and systematic sampling campaigns (e.g., spotlighting and camera‐trap surveys) conducted by the authors (Pople, pers. obs.). We filtered imprecise records (a coordinate uncertainty >1 km) and ensured that this left at least 50% of the dataset and at least 20 unique records per species to maintain an adequate sample size. We removed records when there was more than one within a 1 km^2^ cell. This resulted in records for chital (*n* = 359), fallow (D*ama dama*; *n* = 7013), red (*Cervus elaphus*; *n* = 16,263), sambar (*C. unicolor*; *n* = 869), rusa (*C. timorensis*; *n* = 269), and hog (*A. porcinus*; *n* = 79) deer. For modeling, we selected 20 environmental variables (e.g., bioclimatic, topographic, soil, and geological) from the literature likely to be important predictors of deer distributions in Australia. Bioclimatic variables (i.e., temperature seasonality, maximum temperature of warmest month, minimum temperature of coldest month, annual precipitation, precipitation seasonality, precipitation of wettest quarter, and precipitation of driest quarter) were sourced from WorldClim (Fick & Hijmans, 2017), while dominant lithology (Hartmann & Moosdorf, 2012), vegetation layers (i.e., FAPAR mean and FAPAR seasonality; Copernicus Land Monitoring Service, 2018), landcover (ESA, 2017), and topographic ruggedness and soil properties (i.e., organic carbon, phosphorus content, soil pH, soil bulk density, soil type; FAO/IIASA/ISRIC/ISSCAS/JRC, 2012) were obtained from various sources. Distance to freshwater was derived from HydroSHEDS (https://www.hydrosheds.org/). For detailed information, see Table S1. These variables were global raster layers that were sourced at 1 km resolution, generalized from a finer resolution raster, or rasterized from detailed vector data. For the niche overlap methods, we removed all predictor variables that were highly correlated (Pearson correlation coefficient >.80) to reduce multicollinearity. We were left with 16 variables (Table S2). For MaxEnt modeling, the entire full set of global environmental rasters (20 variables) were used, regardless of collinearity.

### 2.2 Niche overlap methods

To estimate climatic niche overlap between the native and Australian ranges of the six deer species, we used the *ecospat* package (Broennimann et al., 2021; R Studio Team, 2017). This method uses principal component analyses calibrated on the whole environmental space in both the native and exotic ranges. This allows plotting of kernel‐smoothed density estimates of occurrence records in the principal component space to quantify the differences between native and invaded niches using Schoener's *D* index, which varies from 0 (complete dissimilarity) to 1 (complete overlap; Broennimann et al., 2012; Di Cola et al., 2017).

We produced niche overlap plots comparing the deer's international (all records outside of Australia) and Australian niches using species records and environmental variables. To investigate how the six deer species in Australia exhibited niche shifts between international and Australian ranges, we calculated a kernel density distribution map of each species' occurrence records (Di Cola et al., 2017). For each of the deer species in Australia, we compared the environmental conditions available in their international and Australian ranges. We created occurrence density models and determined the contribution of different environmental variables to species distributions. We then tested for niche similarity between each set of compared ranges by randomizing the occurrence records and calculating Schoener's *D* 1000 times each. Next, we compared the observed values with the null distribution of values (i.e., the randomized occurrence records; Broennimann et al., 2012; Da Re et al., 2020). If the observed value fell within this range, we assumed that the ranges were no more similar than random. In contrast, if the value was significantly (*p* < .05) distant from the mean of the null model, the international and Australian ranges were similar. We used the niche similarity test to assess both niche shifts and the niche conservatism (i.e., how similar the niches are between the native and invaded ranges) of the six deer species in Australia (Srivastava et al., 2020).

We calculated niche stability, niche expansion, and the unfilled niche for each deer species. Niche stability represents the proportion of one niche that has conditions identical to another range (i.e., determining whether species occupy identical environmental space in both ranges). In contrast, niche expansion represents the non‐overlapping environmental space between ranges (i.e., determining if species occur in novel environmental conditions not found in their native range; Petitpierre et al., 2012). Finally, an unfilled niche represents the proportion of occurrence records in one range that are present in unused environments in another range (i.e., if a species only partially fills its potential environmental niche in an invaded range; Polidori et al., 2018).

### 2.3 Maxent modeling methods

To model habitat suitability for each of the six deer species in Australia, we constructed species distribution models using maximum entropy (MaxEnt V. 3.4.0) modeling. MaxEnt uses occurrence records and “background” data points to estimate the probability of the presence of a species, generating an index of suitable habitat from 0 (lowest suitability) to 1 (highest suitability; Elith et al., 2011; Philips et al., 2006). We used a target background that is based on known occurrences of similar species. Because Australia has no native deer, we used global records of deer and Australian records of macropods (Macropodidae), buffalo (*Bubalis bubalus*), and goats (*Capra hircus*). As such, we used macropods as the Australian native herbivore, and buffaloes and goats as widespread invasive browser/grazer equivalents. We used the world as a background due to the global distribution and invasiveness of deer. This type of target background corrects for sampling and detection bias within a group of ecologically similar species recorded using similar sampling methods (Phillips et al., 2009).

To model habitat suitability for the six deer species in Australia, models were trained on all available native and invasive records for each species (including Australia). We used 10‐fold cross‐validation using 10% of the data as test data and 90% for training. After cross‐validation, we performed variable selection based on each variable's permutation importance (i.e., the estimation of the importance of the variables; Table S2), resulting in models using only variables that had a permutation importance of over 1% for each species. The models were then re‐run, using these variables and 10‐fold cross‐validation. The average area under the receiver operating characteristic curve (AUC; i.e., indications of model performance) was based on this latter run. We then used the “Fixed cumulative value 10” threshold from the MaxEnt output for each species to set a threshold for discriminating suitable from non‐suitable habitat. This threshold was selected in an attempt to capture even marginally suitable habitat, as invasive species can expand their realized niche into non‐suitable areas that would not have been identified using stricter thresholds. Other than using a target background and variable selection, default MaxEnt settings were used. While tuning individual species models is generally advised, we kept the parameters constant to better compare results between our deer species.

To identify dissimilarity in suitability between the native range of each deer species and its range in Australia, we applied multivariate environmental similarity surface analyses (MESS; Elith et al., 2010). MESS analysis allows visualization of the similarity between pixels predicted to be suitable in Australia (as determined from the MaxEnt modeling), compared with conditions at known occurrences in the native range. A positive MESS value represents a pixel where there is high similarity between occupied native habitat and invaded habitat, and a negative value indicates dissimilarity between native and invaded habitats (Broennimann et al., 2014; Elith et al., 2010). Note that MESS is often used to assess the suitability of MaxEnt models to project habitat suitability across novel climates. This is not the case here, as native and invasive ranges were both used for model training. Rather, MESS analyses were used here separately from MaxEnt models to assess dissimilarity between native and invaded environments.

To determine where the six deer species in Australia are likely to spread in the future, we created invasion risk maps derived using the suitable habitat layer (established from the MaxEnt modeling) and current deer ranges. To determine if deer could spread to new areas, we calculated cost distances from known occurrences using the R function accost() from the package *gdistance* (Etten, 2017), which calculates the “accumulated distance” using habitat suitability as a cost surface. This analysis assumes that species are more likely to spread to cells with greater suitability estimates from points of known occurrence. These cost distance values are used to down‐weight values from the initial suitability map such that areas far away and difficult to reach have small invasion risk values even if their initial suitability values are high. We then scaled these values from 0 (very far and hard to invade) to 1 (near known occurrences and high invasion risk). To determine the area in which species are predicted to spread, we removed areas that were already occupied by deer, and this area was calculated using the α‐hull methodology (Burgman & Fox, 2003) in the *alphahull* package (Pateiro‐Lopez & Rodriguez‐Casal, 2019). We applied an α‐hull value of 1.5 to all species. Using the Australian occurrence records of each species, we generated maps of each deer's present range and overlaid those with maps of invasion risk (i.e., removed the area that was already occupied by the deer).

## 3 RESULTS

### 3.1 Niche shifts

**FIGURE 1:**
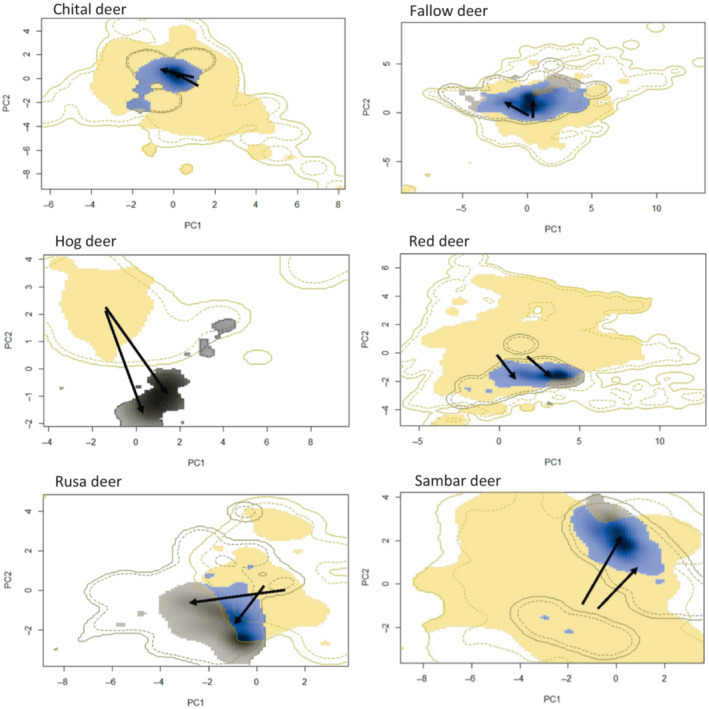
Niche overlap (blue) of deer between their international range (tan) and Australian range (dark brown). The international range was calculated using all records outside Australia. The Australian range was modeled using records from Australia only. In all plots, blue areas represent the overlap between the different niches. Darker patches represent the highest population density in both ranges, and solid and dashed contour lines illustrate 100% and 50% of the available environmental space, respectively. Arrows visualize the shift of the centroids between respective distributions.

We found evidence of niche shifts in some species, with low niche overlap values when we made pairwise comparisons of each species' international and Australian ranges (*D* = 0–0.292; Table 2; Figures S1–S6). Hog, rusa, sambar, and fallow deer exhibited relatively large niche shifts, and thus underwent significant niche expansion following introduction to Australia (Figure 1; Table 2). In contrast, the ranges of chital and red deer in Australia are enclosed within the total niche envelope of their international ranges, exhibiting limited niche expansion (0.007 and 0.134, respectively) and high stability (0.993 and 0.866, respectively). Despite this, niche similarity between international and Australian ranges of red deer was still not statistically significant (*p* = .150). There was significant similarity between the international and Australian niches of chital deer (*p* < .001). Fallow deer also exhibited relatively high niche stability (0.892; Table 2) although, unlike chital or red deer, fallow deer had some degree of niche expansion (Table 2). Hog deer exhibited no niche overlap between international and Australian ranges (Figure 1c) and thus showed no niche stability (0.000) and high niche expansion (1.000).

**TABLE 2:** Results of equivalency and similarity testing for niche overlap of the international and Australian distributions of each of the six deer species in Australia.

	Schoener's *D*	Similarity	Expansion	Stability	Unfilled
Chital deer	0.087	**0.010**	0.007	0.993	0.799
Fallow deer	0.292	0.061	0.108	0.892	0.337
Hog deer	0.000	1.000	1.000	0.000	1.000
Red deer	0.041	0.150	0.134	0.866	0.725
Rusa deer	0.317	0.060	0.715	0.285	0.799
Sambar deer	0.064	0.231	0.082	0.918	0.968

*Note*: Bold indicates significant niche similarity between international and Australian ranges.

Species niche profiles for each variable were also quantified (Figures S7–S12), with maximum temperature in the warmest month, minimum temperature in the coldest month, and average annual rainfall selected to compare international and Australian ranges of deer (Table 3). While most species still occur across a broad climatic range in Australia, the six species differed in the direction of their niche shifts. Fallow deer have spread into warmer niches in Australia compared to other parts of their international range. Hog and rusa deer shifted into drier and colder ranges following introduction to Australia. Red deer shifted to wetter and warmer areas, and sambar deer are present in areas colder than those experienced in their international ranges. In contrast to the other five species, chital deer in Australia still inhabit niche profiles very similar to their international range (although the Australian range is drier on average).

**TABLE 3:** Difference between the tested niche overlap variables of the international and Australian ranges (i.e., international values minus Australian values) for the six free‐living deer species in Australia.

	Difference		
	Average annual rainfall (mm)	Average maximum temp. (°C)	Average minimum temp. (°C)
Chital deer	534.05	2.46	0.93
Fallow deer	−80.77	−4.04	−4.86
Hog deer	1228.93	8.76	5.67
Red deer	−135.99	−4.29	−8.85
Rusa deer	502.45	3.28	9.55
Sambar deer	349.70	11.11	11.82

### 3.2 Habitat suitability modeling and present ranges

**FIGURE 2:**
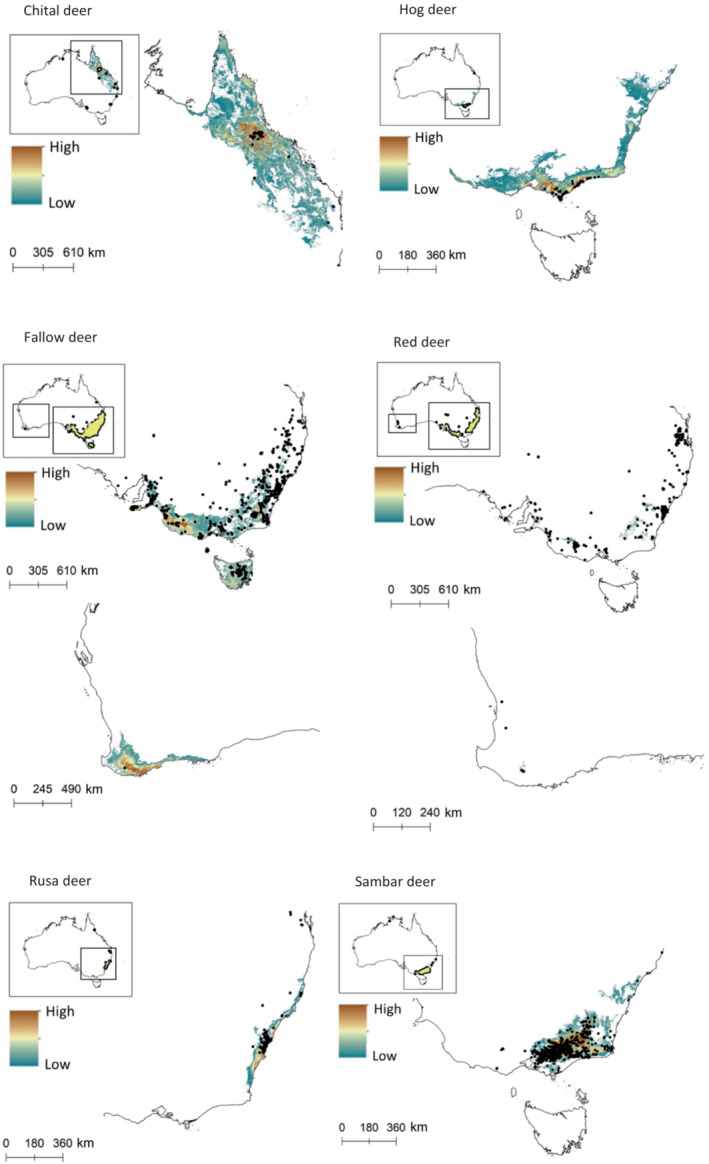
Maps of invasible habitat and future spread range from high vulnerability (brown) to low vulnerability (teal) as determined by MaxEnt modeling, including records (dots) of the six feral deer species in Australia. For maps including the present range polygons, see Figure S14.

**FIGURE 3:**
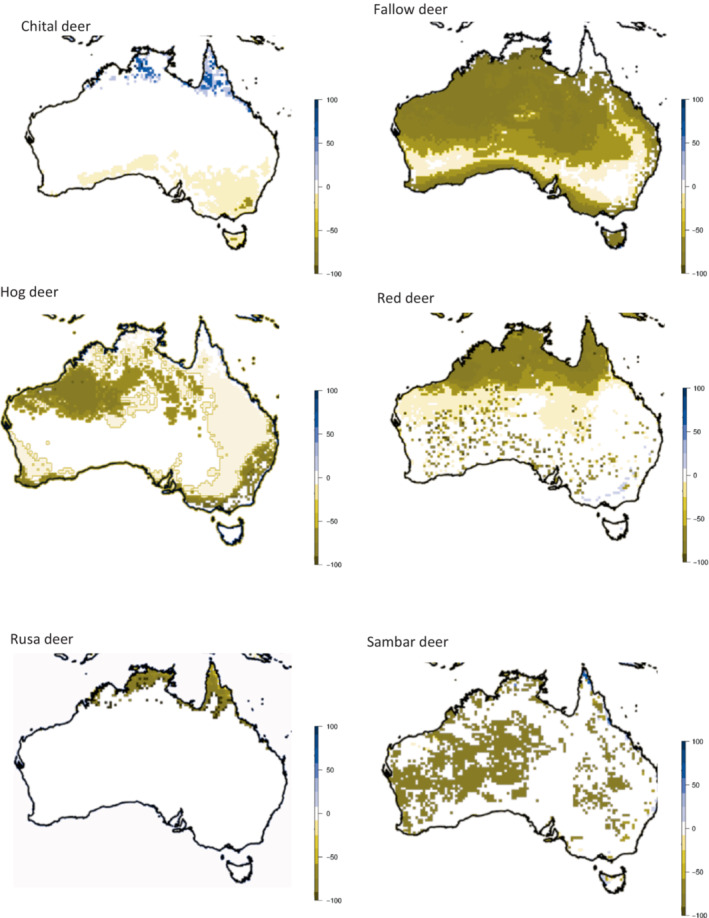
Habitat suitability predicted from native and introduced ranges of the six deer in Australia. Symbology bar indicates similarity with dark blue indicating high similarity (100%) and dark brown indicating low (−100%).

Our models predicting the future suitable habitat of deer in Australia performed well; AUC values for all species were >0.85 (Figure S13). Contributing variables are also presented in Table S3. Of all deer species examined here (Figure 2), chital and hog deer had large potentially suitable areas that have not yet been invaded, leading to high percentage differences between areas of suitable habitat and areas that have not yet been invaded (4790% and 1443%, respectively). Results of the MESS analyses (Figure 3) demonstrated that of the six deer species in Australia, chital deer had the largest area of habitat that was most similar to their native range. Fallow deer had the largest area of uninvaded suitable habitat (123,665 km^2^) but, because they have invaded such a large area already, this only represented 19% of the area that is presently occupied (654,193 km^2^). Rusa deer have a relatively small area of potentially suitable habitat not yet invaded (18,668 km^2^), however, this represented a 73% increase from the area that they presently occupy. Red and sambar deer both have much smaller areas of uninvaded but potentially suitable habitat (2% and 16%, respectively, of their presently occupied areas). Maps of potentially suitable area with present distributions overlaid are provided in Figure S14.

## 4 DISCUSSION

Five of the six deer species introduced to Australia (fallow, hog, red, rusa, and sambar deer) occupy significantly different niches in Australia compared to their international niche profiles (Figure 1). Range estimate models suggest that fallow, red, and sambar deer have already spread beyond habitats typical of their international niches. In contrast, chital, hog, and rusa deer have the potential to spread much further than their present distributions. Of all the species examined, chital deer have the greatest predicted range in Australia, orders of magnitude greater than the other species. As such, chital deer potentially represent a management problem following invasion in northern and eastern Australia.

In the future, deer in Australia are likely to expand their current distributions (Figure 2). Fallow deer are currently spreading north from their current distributions in Victoria and New South Wales, beyond habitat predicted to be suitable, and establishing in areas that are warmer than the international range. Likewise, red deer are present in areas that are warmer, but also wetter than where they occur outside Australia. Hog and rusa deer have shifted into drier and colder ranges following introduction to Australia. Hog deer have a high degree of potentially invasible habitat north of their current distribution in Victoria, and it seems likely that they will spread into this area. Rusa deer are expanding south along the south‐eastern coast of Australia. Finally, sambar deer are predicted to spread further north along the north‐eastern coast from their present range. Many of these invasible areas represent valuable agricultural and conservation areas. Deer in Australia are already competing with livestock for forage and feeding on crops (Bentley, 1998; Davis et al., 2016). Deer invasions into natural areas are likely to cause degradation of water quality through trampling, erosion, and increased nutrient loading (McDowell, 2007, 2008). Impacts of deer are likely to increase in these sectors as deer populations continue to grow and spread beyond their present distributions.

As populations increase in size, genetic variation should also increase, which facilitates evolution and adaptation to new environments (Lee, 2002; Urban et al., 2007). Likewise, as yet unrealized phenotypic plasticity could allow populations of invasive species to expand quickly (Davidson et al., 2011). Population expansion can drive individuals into suboptimal habitats, thus forcing animals to face novel environmental conditions (Hardie & Hutchings, 2010; Urban et al., 2007). Even without adaptation, chital, hog, and rusa deer have the capacity to spread beyond their current distributions. Considering the ability of these species to exhibit niche shifts, it is likely that the species with currently limited distributions will expand their environmental niches in the future. Once chital, hog, and rusa deer in Australia have filled their potentially suitable habitats, they may adapt and expand beyond their respective ranges, much like fallow, red, and sambar deer.

Based on our models, chital deer have the capacity to spread further from their present distribution than the other five species in Australia (Table 4). There were no significant differences between niches in the international and Australian ranges for chital deer, probably because they were introduced to habitat similar to their native range (Figure 3a). Compared to the other deer species in Australia, chital deer have not had to adjust to a particularly novel environment (Figure 3). Since their present distribution is relatively restricted compared with other deer species in Australia, population spread may be limited by other biotic or abiotic variables (Kelly et al., 2021; Watter et al., 2019). Despite this, chital deer represent a significant risk in the Australian environment, because much of the present habitat adjacent to their current distribution is ecologically similar to their native range. They are likely to spread even without any intrinsic changes in their habitat requirements.

**TABLE 4:** Total area (km^2^) presently occupied (present range) and uninvaded habitat, calculated from Figure 2.

	Present range	Uninvaded	% Difference
Chital deer	10,667	510,957	4790
Fallow deer	654,193	123,655	19
Hog deer	6916	99,806	1443
Red deer	262,287	5188	2
Rusa deer	25,657	18,668	73
Sambar deer	101,957	15,953	16

*Note*: The % difference represents the area between the present range and the threatened range that has not yet been invaded.

In contrast to chital deer, the other five species of deer in Australia have exhibited significant niche shifts since arriving. As many of the deer species in Australia have broad international ranges (except for hog deer), international ranges likely represent something akin to their fundamental niche, and the observed spread into new niche space in the Australian environment likely represents true niche shifts. Many invasive species undergo rapid evolution following invasion, quickly adapting to conditions in the novel environment (Broennimann et al., 2007; Callaway & Maron, 2006; Maron & Alexander, 2014), which we believe has likely occurred in several deer species introduced into Australia.

Hog deer have a very limited history of introduction worldwide, and their invaded range is almost completely confined to Australia (Hill et al., 2019). Prins and Gordon (2014) proposed that a species will not invade areas with abiotic conditions outside its physiological tolerance levels. If we accept this theory, then these Australian ecosystems must fall within their physiological tolerance. The success of hog deer in Australia demonstrates that species with limited native or worldwide distributions can spread beyond predicted ranges, simply because we do not know their physiological tolerances. In addition, physiological tolerances can evolve (Lee et al., 2003; Qu & Wiens, 2020) and invasive species can exhibit a high degree of phenotypic plasticity (Davidson et al., 2011). Even with accurate knowledge of physiological tolerances, the accuracy of predictions of spread may be limited.

While habitat suitability has certainly contributed to the success and spread of invasive deer in Australia, the number of deer introductions, or propagule pressure, has also likely played a role (Fautley et al., 2012; Forsyth et al., 2004). Propagule pressure influences establishment success, as well as subsequent viability of a population (Forsyth & Duncan, 2001; Leung et al., 2004; Lockwood et al., 2005; Prins & Gordon, 2014). The chital deer population in North Queensland arose from four individuals released in 1886, and the hog deer founding population comprised 15 individuals in Victoria, with no subsequent releases (Bentley, 1967; Hill et al., 2019; Moriarty, 2004). Interestingly, chital and hog deer have spread the least from their point of liberation (occupying 10,667 and 6916 km^2^, respectively) compared to the other four species in Australia (ranging from 25,657 [rusa] to 654,193 km^2^ [fallow deer]; Table 4), which all experienced multiple introductions (Bentley, 1967). In contrast, species that failed to establish were often introduced a limited number of times. For example, wapiti (*Cervus canadiensis*), Chinese water deer (*Hydropotes inermis*), and Eld's deer (*Cervus eldii*) were reportedly introduced to or escaped from only one location each, while barasingha (*Cervus duvaucelii*) was released twice. While the sample size is low, this pattern is consistent with the hypothesis that species with more introductions now have wider ranges and greater niche shifts.

Previous modeling to estimate the spread of deer in Australia (Davis et al., 2016; Moriarty, 2004) used climate‐matching models that only compare the climate of a species' current geographic range with the climate of a target location (Baker & Bomford, 2009) as opposed to creating more broadly‐based SDMs. Climate‐matching models make simple associations between occurrence localities and climate variables, but SDMs (such as MaxEnt) that use regression or machine‐learning methods can fit more complex responses and thus better capture niche relationships (see Froese, 2012 for a comprehensive comparison). Predicting potential species ranges using climate matching (e.g., CLIMATCH or CLIMEX; Bureau of Rural Sciences, 2008; Sutherst et al., 1999) often occurs on a much coarser scale than species distribution modeling due to the available settings and limited customization of predictor variables, thus overestimating the potential range of invasive species (Elith et al., 2011; Froese, 2012; Kumar et al., 2015; Srivastava et al., 2019; Wearne et al., 2013). Because of the more detailed response, the complexity of MaxEnt for habitat suitability modeling allows more accurate predictions of the future distribution of invasive species, compared with previous methods.

The niches of invasive species are capable of shifting over time as they adapt to novel environments (Fitzpatrick et al., 2007; Jourdan et al., 2021; Morehouse & Tobler, 2013; Parravincini et al., 2015). Deer have had the opportunity to invade, and subsequently adapt to, many areas around the world. As such, we might expect that many deer species have had the opportunity to fill their entire environmental niche. Here we demonstrate that five of the six deer species introduced to Australia showed significant shifts in their environmental niches, and three have already spread beyond their predicted suitable habitat. As deer continue to move into different environments, it is likely that they will continue to adapt to previously unavailable niches, thus increasing their potential for future spread, not only in Australia, but worldwide. If this continues, then these pest species will be far more problematic and widespread than we can predict using SDMs alone.

## AUTHOR CONTRIBUTIONS

**Catherine L. Kelly:** Conceptualization (equal); data curation (equal); formal analysis (equal); investigation (equal); methodology (equal); visualization (equal); writing – original draft (equal); writing – review and editing (equal). **Iain J. Gordon:** Conceptualization (equal); investigation (equal); supervision (equal); writing – original draft (equal); writing – review and editing (equal). **Lin Schwarzkopf:** Conceptualization (equal); supervision (equal); writing – original draft (equal); writing – review and editing (equal). **Anna Pintor:** Data curation (equal); formal analysis (equal); methodology (equal); writing – review and editing (equal). **Anthony Pople:** Data curation (equal); project administration (equal); writing – review and editing (equal). **Ben T. Hirsch:** Conceptualization (equal); supervision (equal); writing – original draft (equal); writing – review and editing (equal).

## FUNDING INFORMATION

This manuscript was partially funded through an ARC Linkage grant to BTH (LP1801000267). We thank DAF Queensland and JCU for additional support.

## CONFLICT OF INTEREST STATEMENT

The authors declare no conflict of interest.

## Supporting information


Appendix S1.

## Data Availability

Occurrence records from ALA and GBIF and environmental data are available on the relevant referenced publicly available databases. Occurrence records from FeralScan were collected and used in agreement with Peter West and FeralScan and are not publicly available.
