# Supplementary material for: Invasive wild deer exhibit environmental niche shifts in Australia: Where to from here?
Source: Ecol Evol. 2023 Jul 3;13(7):e10251. doi: 10.1002/ece3.10251 (PMC10316481; doi:10.1002/ece3.10251)
Supplement: Supplementary file 1 — Appendix S1. [file ECE3-13-e10251-s001.docx]

Supplementary material:

Table 1. Input variables for MaxEnt modelling

| Variable name | Variable description | Source |
| --- | --- | --- |
| BIO4 | Temperature seasonality (standard deviation x 100) | WorldClim V2.0 datasets: https://www.worldclim.org/ |
| BIO5 | Max temperature of warmest month (°C) | “ |
| BIO6 | Min temperature of coldest month (°C) | “ |
| BIO12 | Annual precipitation (mm) | “ |
| BIO15 | Precipitation seasonality (Coefficient of variation) | “ |
| BIO16 | Precipitation of wettest quarter (mm) | “ |
| BIO17 | Precipitation of driest quarter (mm) | “ |
| landcover | Categorical variable | https://www.esa-landcover-cci.org/ |
| FAPAR mean | Average vegetation greenness | https://land.copernicus.eu/global/products/fapar |
| FAPAR seasonality | Seasonality in vegetation greenness | https://land.copernicus.eu/global/products/fapar |
| freshwater distance | Distance to freshwater (m) | https://www.esa-landcover-cci.org/ & https://www.hydrosheds.org/ |
| topographic ruggedness | Measure of topographic ruggedness | https://www.fao.org/soils-portal/data-hub/soil-maps-and-databases/harmonized-world-soil-database-v12/en/ |
| water cap | Soil water capacity | https://www.isric.org/explore/wise-databases: |
| organic carbon | Soil organic carbon | “ |
| soil pH | Soil pH | “ |
| clay | Percent clay in soil (%) | https://www.fao.org/soils-portal/data-hub/soil-maps-and-databases/harmonized-world-soil-database-v12/en |
| soil bulk density |  | https://www.isric.org/explore/wise-databases |
| soil type | Categorical variable | https://www.fao.org/soils-portal/data-hub/soil-maps-and-databases/harmonized-world-soil-database-v12/en |
| phosphorus | Soil phosphorus retention class | https://www.isric.org/explore/wise-databases |
| lithology | dominant lithology | https://agupubs.onlinelibrary.wiley.com/doi/full/10.1029/2012GC004370: |

Table 2. Important variables as determined by MaxEnt modelling for each of the six species of deer in Australia. RC = relative contribution to model, PI =permutational importance. In bold are the two most important variables that contribute to habitat suitability for each species.

|  | Chital | | Fallow | | Hog | | Red | | Rusa | | Sambar | |
| --- | --- | --- | --- | --- | --- | --- | --- | --- | --- | --- | --- | --- |
| Variable | RC | PI | RC | PI | RC | PI | RC | PI | RC | PI | RC | PI |
| precipitation seasonality | **21.6** | 7.5 | 2.5 | 14.3 | 7.3 | 11.2 | 3.6 | 8.8 | 13.4 | 37.3 | 7 | 9.1 |
| maximum temperature | **19.4** | 5.5 | **30.2** | 25.2 |  |  | 3.3 | 4.5 | 8.8 | 21.6 | 1.9 | 1.3 |
| soil type | 13.7 | 6.4 | 14.1 | 5.1 | **16.4** | 3 | **14.9** | 8.4 | 15.4 | 2 | 9.2 | 3.9 |
| minimum temperature | 10.5 | 55 | **15.3** | 21.1 | **39.4** | 49.9 | 14.3 | 20.5 | 7.4 | 14.4 |  |  |
| precipitation of driest quarter | 9.5 | 5 | 3.4 | 13.3 | 12.4 | 9.7 | 1.4 | 12.4 | 8.7 | 1.6 | 3.7 | 4.4 |
| annual precipitation | 9 | 9.6 | 0.1 | 1.8 | 12.7 | 25.4 | 1.1 | 9 | **18.2** | 4.3 | 3.3 | 7.7 |
| land cover type | 7.5 | 1.7 | 6.5 | 1.7 | 11.7 | 0.8 | 32 | 16.6 | **24.2** | 2.9 | **31.3** | 14 |
| dominant lithology | 2.3 | 0.4 |  |  |  |  |  |  |  |  | 2 | 1 |
| soil pH | 1.5 | 1.4 |  |  |  |  |  |  | 0.4 | 5.1 |  |  |
| soil phosphorous retention | 1.5 | 0.1 |  |  |  |  | 1.7 | 1.6 |  |  | 4.4 | 1.1 |
| average vegetation greenness | 1.1 | 1.1 | 17 | 4.7 |  |  |  |  | 0.9 | 2 | 3 | 3.8 |
| soil organic carbon | 0.9 | 2.5 |  |  |  |  |  |  |  |  | 0.7 | 3.3 |
| percent clay in soil | 0.7 | 0.9 |  |  |  |  |  |  |  |  | 9.2 | 13.2 |
| soil water capacity | 0.7 | 3 | 7.8 | 6.1 |  |  |  |  |  |  | 0.5 | 3.9 |
| precipitation of wettest quarter |  |  | 2 | 5.7 |  |  |  |  | 0.2 | 5.9 | 0.4 | 4.3 |
| distance to freshwater |  |  | 1.1 | 1.2 |  |  |  |  |  |  | 1.1 | 2.7 |
| temperature seasonality |  |  |  |  |  |  | 0.8 | 14.2 | 2.3 | 2.8 | 8.3 | 13.2 |
| topographic ruggedness |  |  |  |  |  |  | **26.8** | 4 |  |  | **9.4** | 2.5 |
| soil bulk density |  |  |  |  |  |  |  |  |  |  | 3.1 | 4.9 |
| seasonality in vegetation greenness |  |  |  |  |  |  |  |  |  |  | 1.4 | 5.7 |

Table 3. Input variables for niche shift modelling

| Variable name | Variable description | Source |
| --- | --- | --- |
| BIO4 | Temperature seasonality (standard deviation x 100) | WorldClim V2.0 datasets: https://www.worldclim.org/ |
| BIO5 | Max temperature of warmest month (°C) | “ |
| BIO6 | Min temperature of coldest month (°C) | “ |
| BIO12 | Annual precipitation (mm) | “ |
| BIO15 | Precipitation seasonality (Coefficient of variation) | “ |
| BIO16 | Precipitation of wettest quarter (mm) | “ |
| BIO17 | Precipitation of driest quarter (mm) | “ |
| landcover | Categorical variable | https://www.esa-landcover-cci.org/ |
| FAPAR mean | Average vegetation greenness | https://land.copernicus.eu/global/products/fapar |
| freshwater distance | Distance to freshwater (m) | https://www.esa-landcover-cci.org/ & https://www.hydrosheds.org/: |
| topographic ruggedness | Measure of topographic ruggedness | https://www.fao.org/soils-portal/data-hub/soil-maps-and-databases/harmonized-world-soil-database-v12/en/ |
| water cap | Soil water capacity | https://www.isric.org/explore/wise-databases: |
| organic carbon | Soil organic carbon | “ |
| soil pH | Soil pH | “ |
| clay | Percent clay in soil (%) | https://www.fao.org/soils-portal/data-hub/soil-maps-and-databases/harmonized-world-soil-database-v12/en |
| phosphorus | Soil phosphorus retention class | https://www.isric.org/explore/wise-databases |
| lithology | dominant lithology | https://agupubs.onlinelibrary.wiley.com/doi/full/10.1029/2012GC004370: |


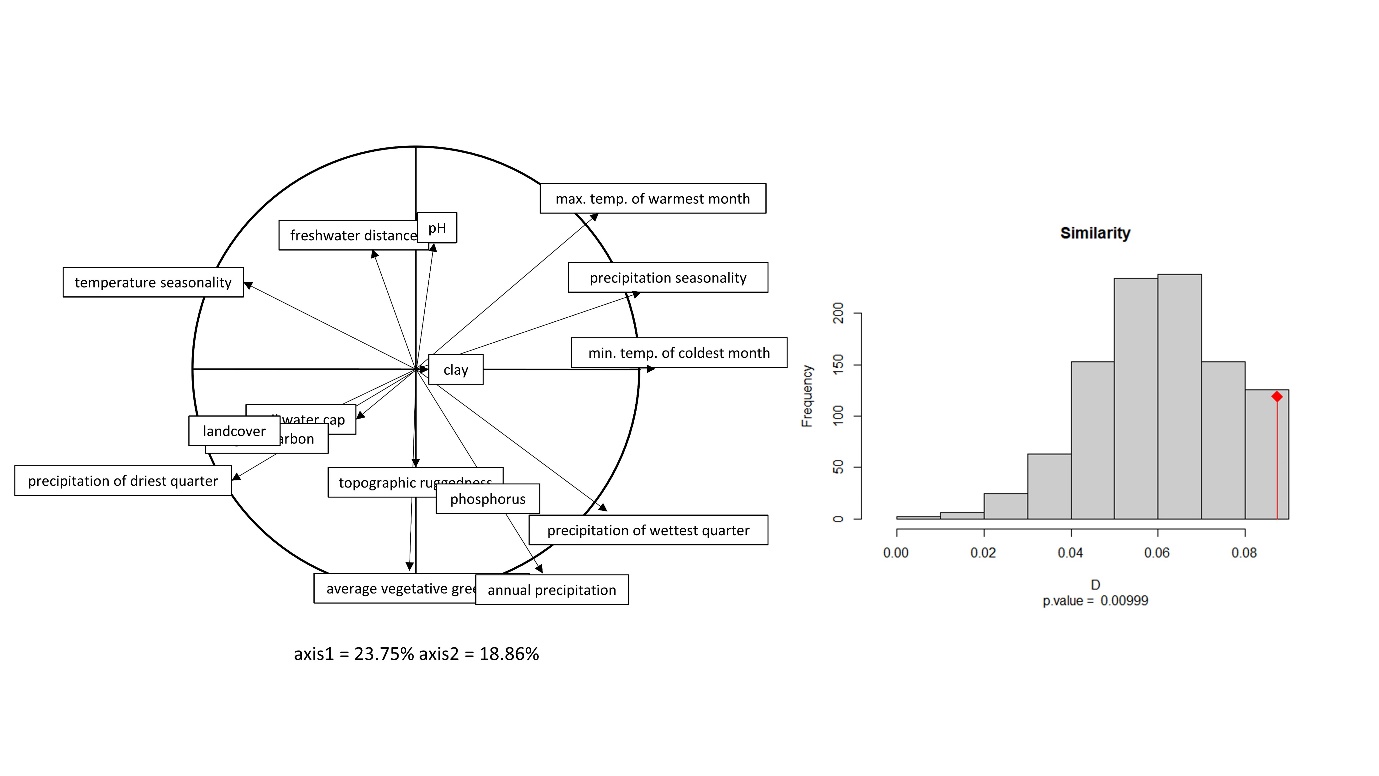


Figure 1. Niche of chital deer in both their total range and Australia. The left correlation circle represents the contribution of each variable along the first two principal axes. Right represents the histogram for the niche similarity test between Australian and international ranges with 1000 replications, with the Schoener’s D (niche overlap index) represented by the red line.


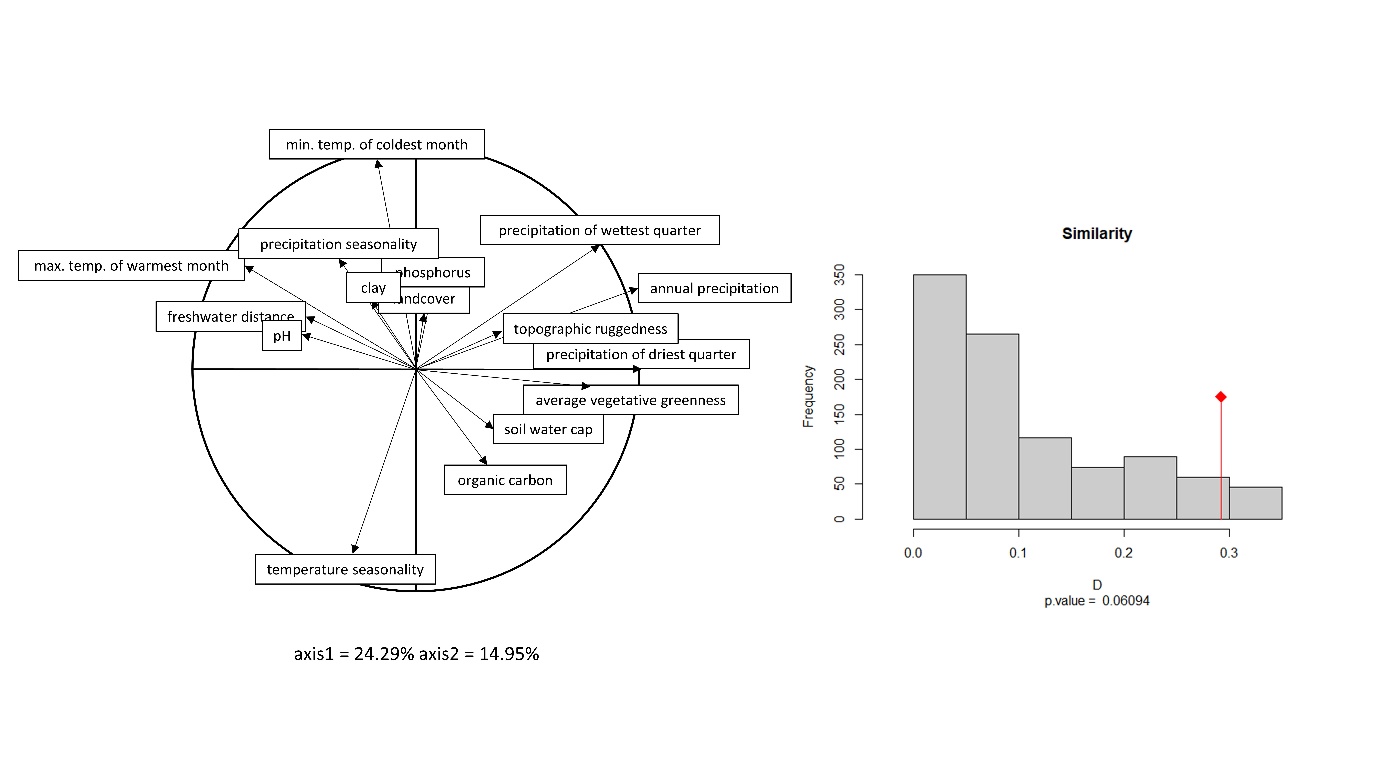


Figure 2. Niche of fallow deer in both their total range and Australia. The left correlation circle represents the contribution of each variable along the first two principal axes. Right represents the histogram for the niche similarity test between Australian and international ranges with 1000 replications, with the Schoener’s D (niche overlap index) represented by the red line.


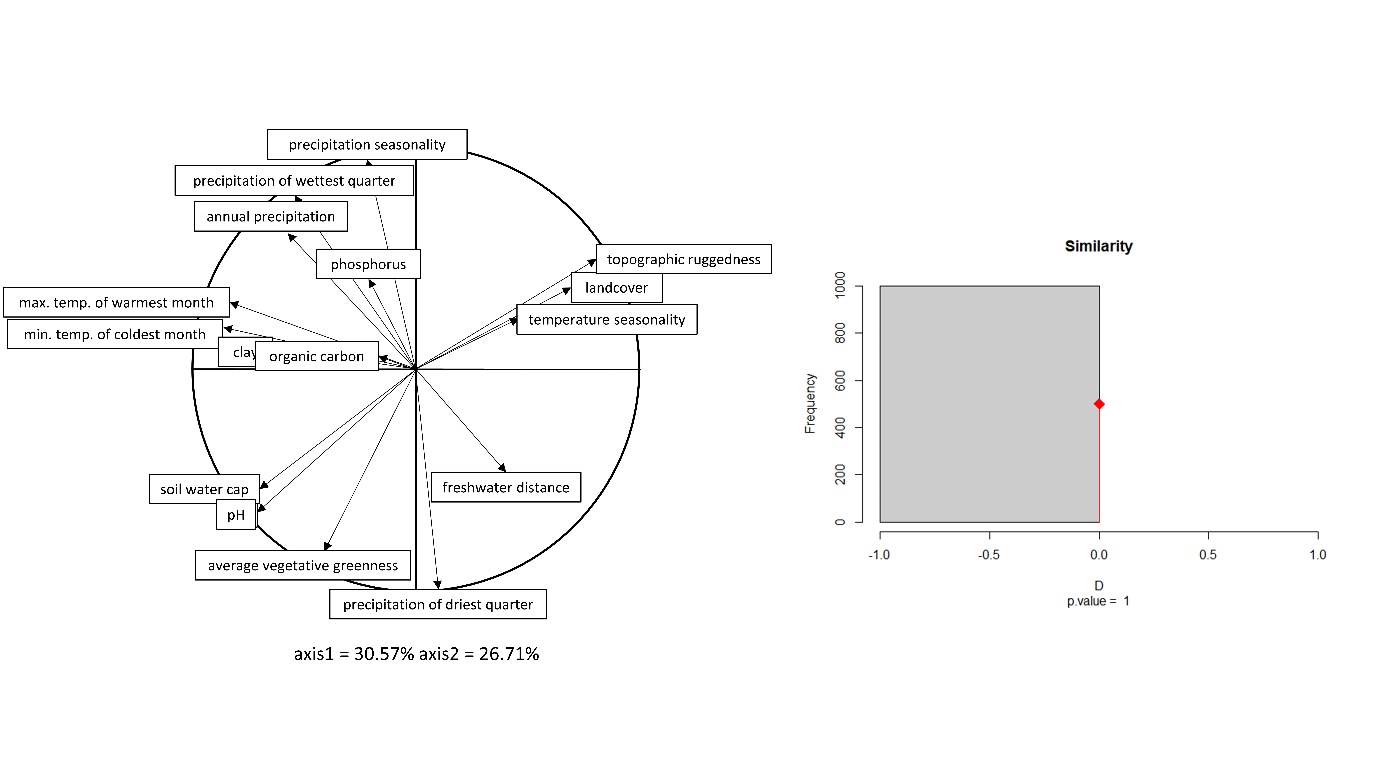


Figure 3. Niche of hog deer in both their total range and Australia. The left correlation circle represents the contribution of each variable along the first two principal axes. Right represents the histogram for the niche similarity test between Australian and international ranges with 1000 replications, with the Schoener’s D (niche overlap index) represented by the red line.


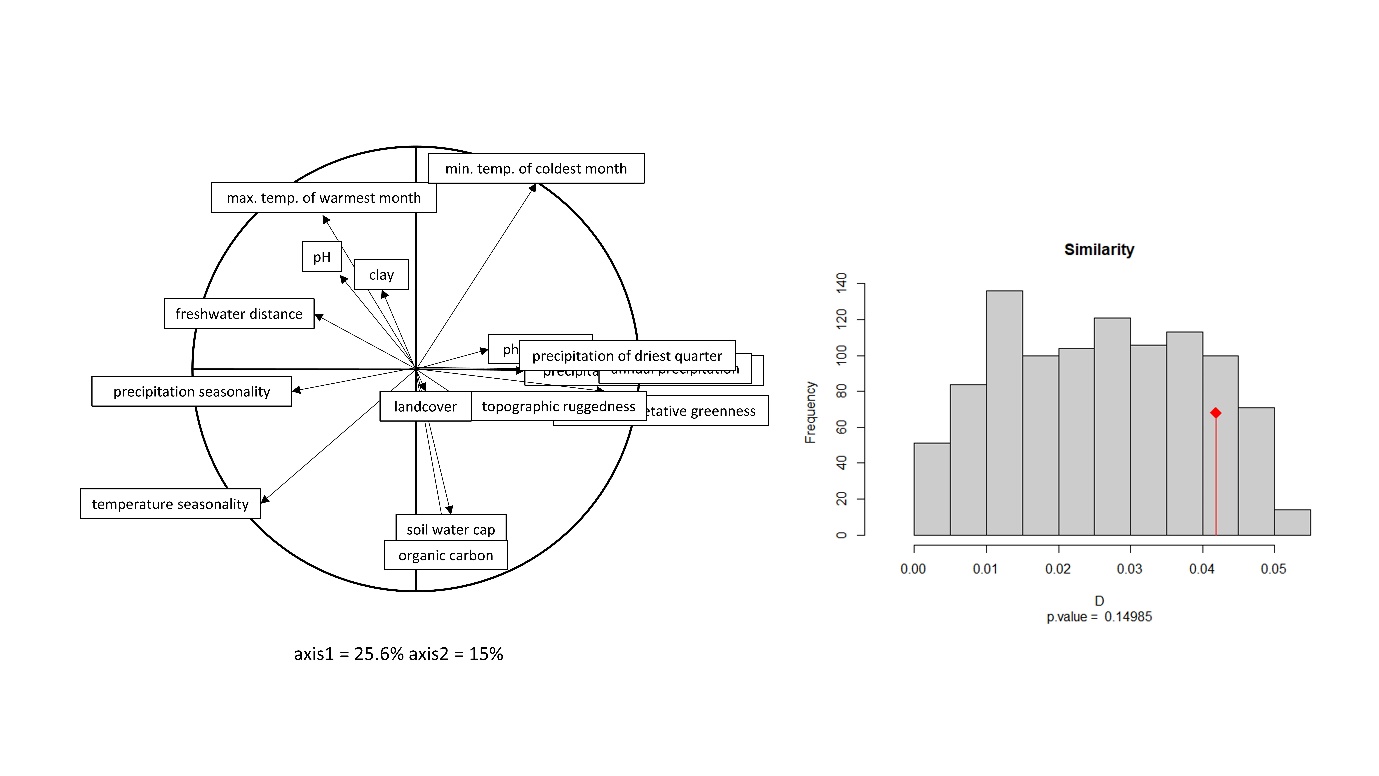


Figure 4. Niche of red deer in both their total range and Australia. The left correlation circle represents the contribution of each variable along the first two principal axes. Right represents the histogram for the niche similarity test between Australian and international ranges with 1000 replications, with the Schoener’s D (niche overlap index) represented by the red line.


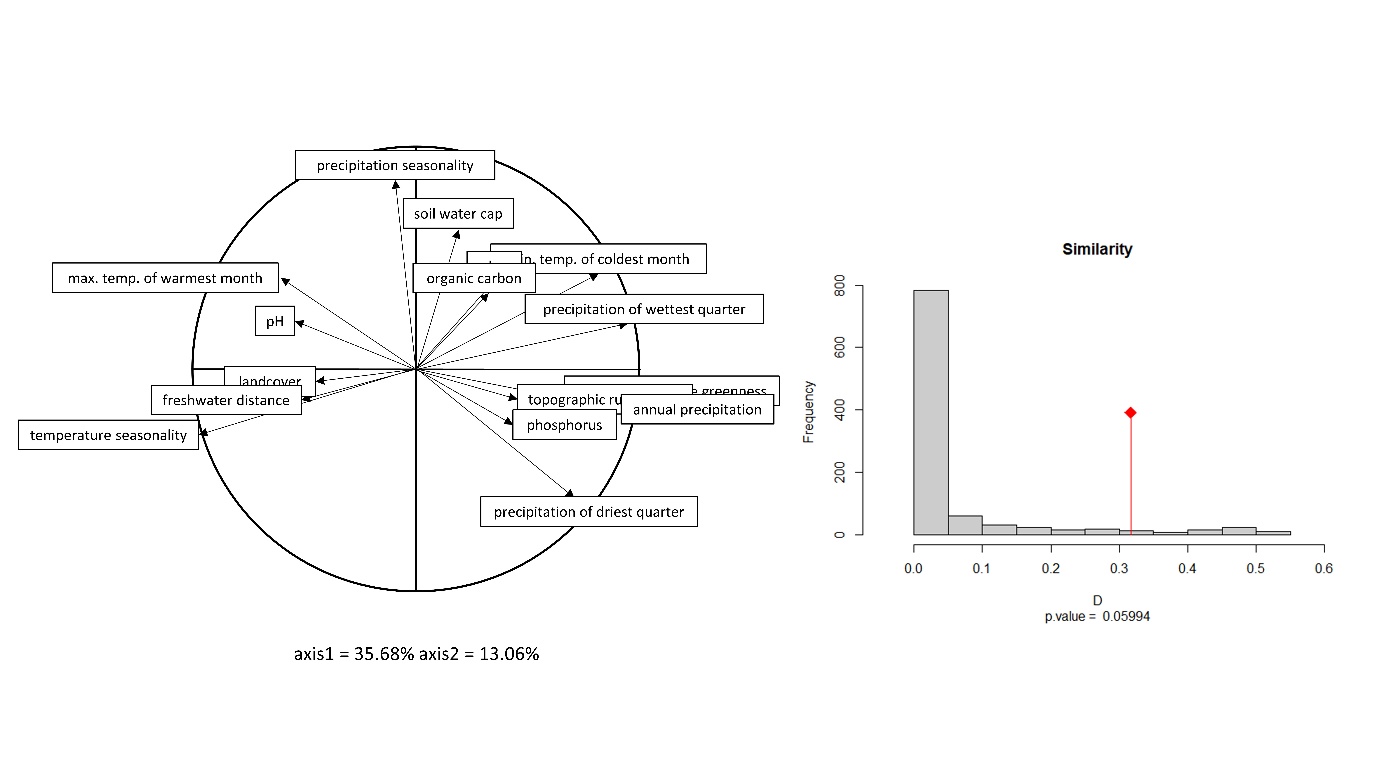


Figure 5. Niche of rusa deer in both their total range and Australia. The left correlation circle represents the contribution of each variable along the first two principal axes. Right represents the histogram for the niche similarity test between Australian and international ranges with 1000 replications, with the Schoener’s D (niche overlap index) represented by the red line.


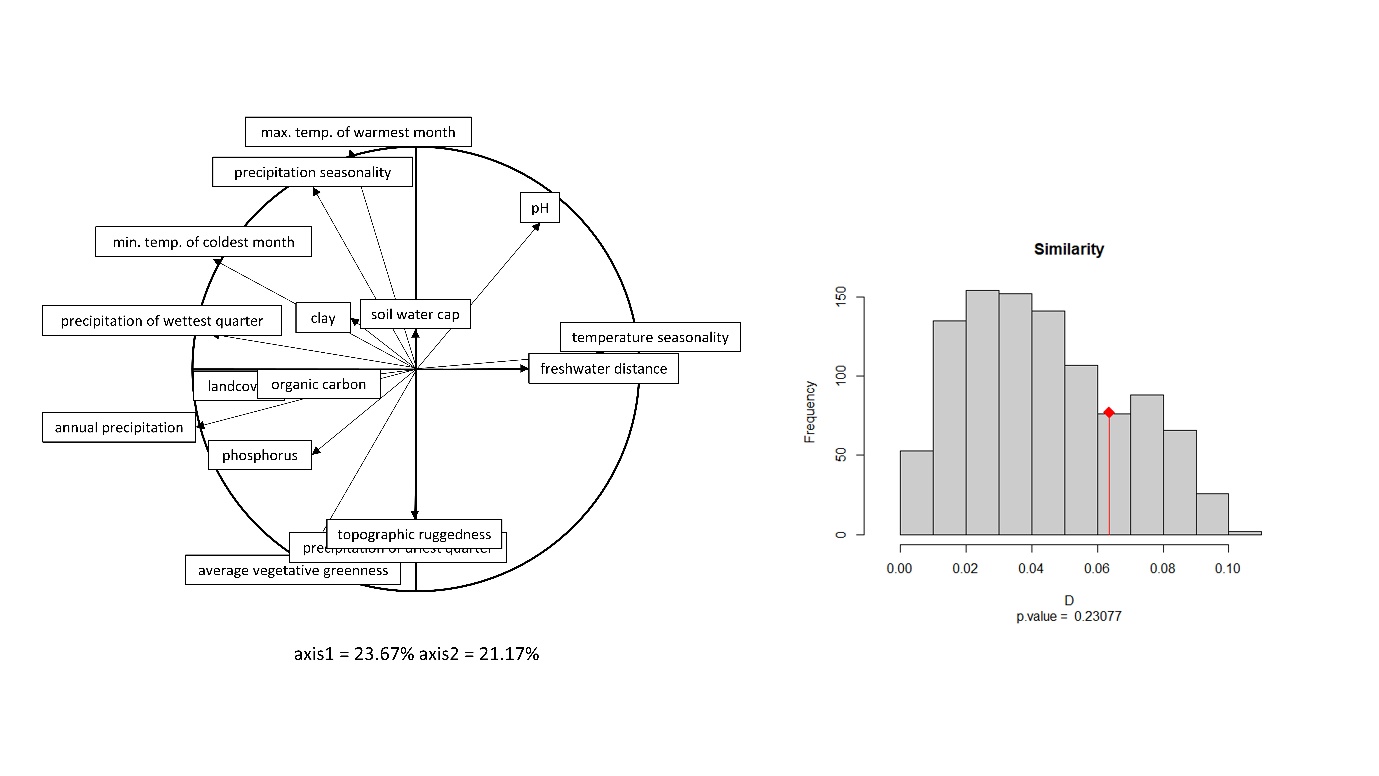


Figure 6. Niche of sambar deer in both their total range and Australia. The left correlation circle represents the contribution of each variable along the first two principal axes. Right represents the histogram for the niche similarity test between Australian and international ranges with 1000 replications, with the Schoener’s D (niche overlap index) represented by the red line.


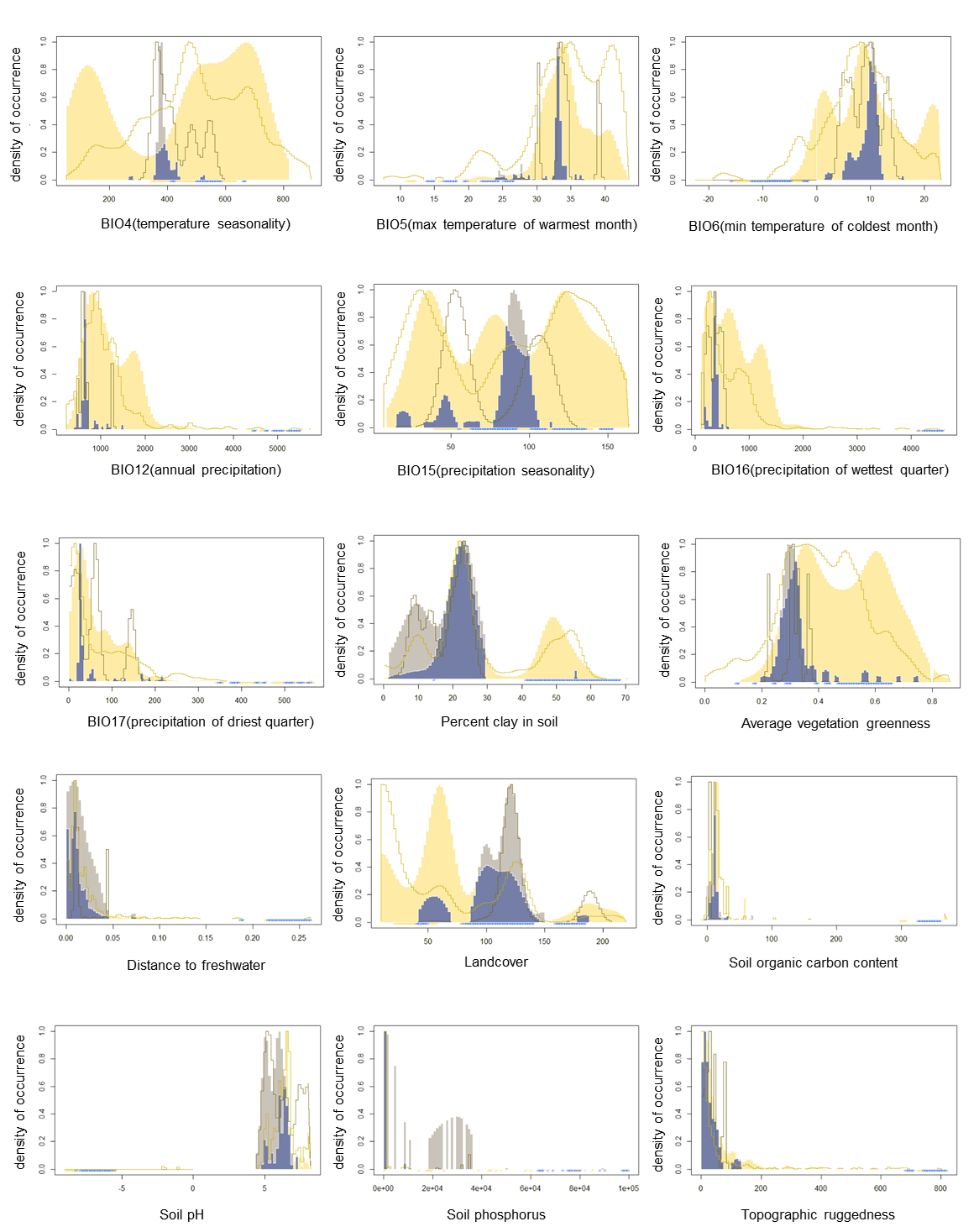


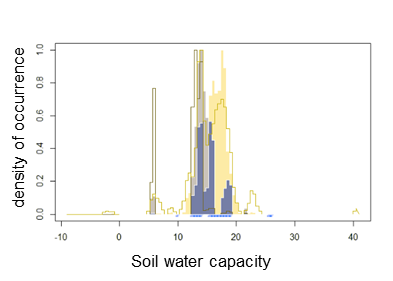


Figure 7. Chital deer niche occupancy profiles for each variable. Tan represents the total range, and dark brown represents the Australian range. Purple represents the overlap between the two ranges. Overlapping peaks indicate similar variable tolerances. Tan solid lines represent 100% of available space in the total range (without Australia) and dark brown represents 100% of the available space in the Australian range. Descriptions of variables used, including units, are provided in Table 1.


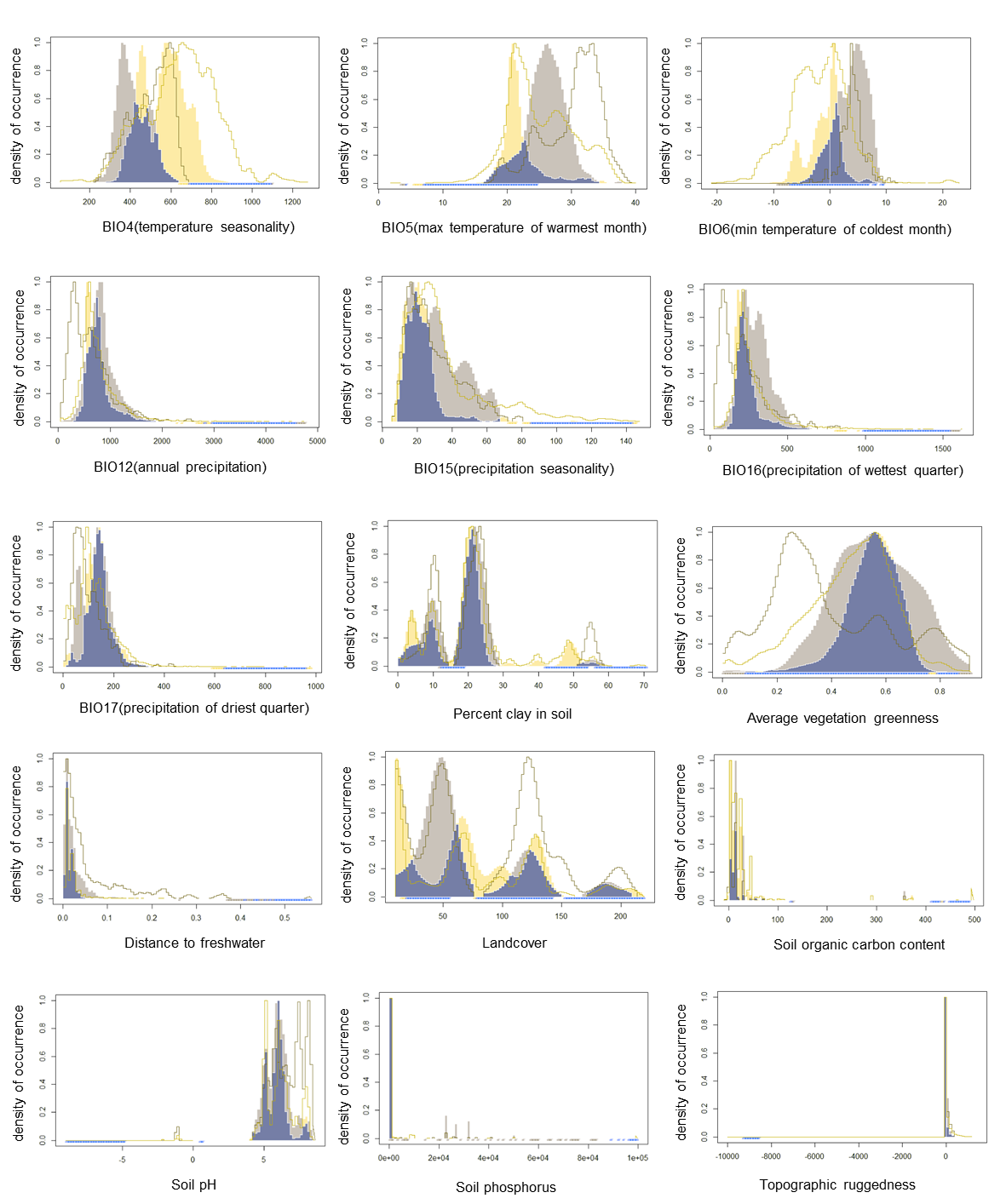


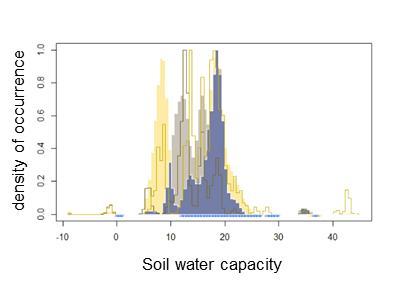


Figure 8. Fallow deer niche occupancy profiles for each variable. Tan represents the total range, and dark brown represents the Australian range. Purple represents the overlap between the two ranges. Overlapping peaks indicate similar variable tolerances. Tan solid lines represent 100% of available space in the total range (without Australia) and dark brown represents 100% of the available space in the Australian range. Descriptions of variables used, including units, are provided in Table 1.


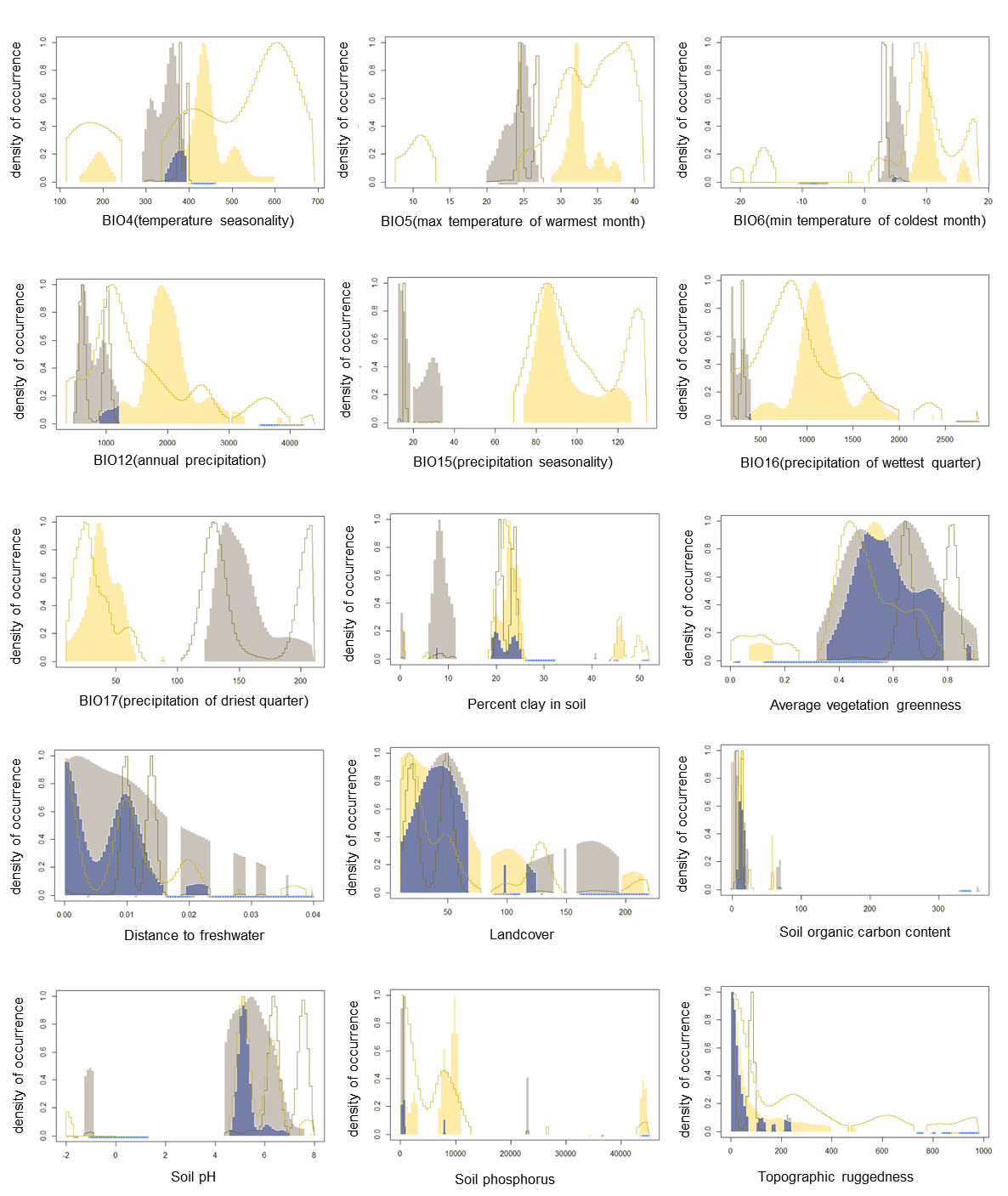


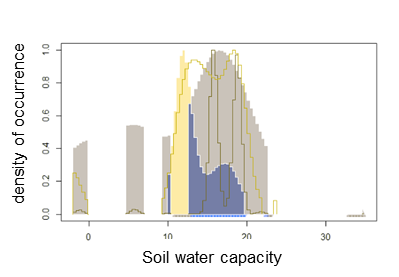


Figure 9. Hog deer niche occupancy profiles for each variable. Tan represents the total range, and dark brown represents the Australian range. Purple represents the overlap between the two ranges. Overlapping peaks indicate similar variable tolerances. Tan solid lines represent 100% of available space in the total range (without Australia) and dark brown represents 100% of the available space in the Australian range. Descriptions of variables used, including units, are provided in Table 1.


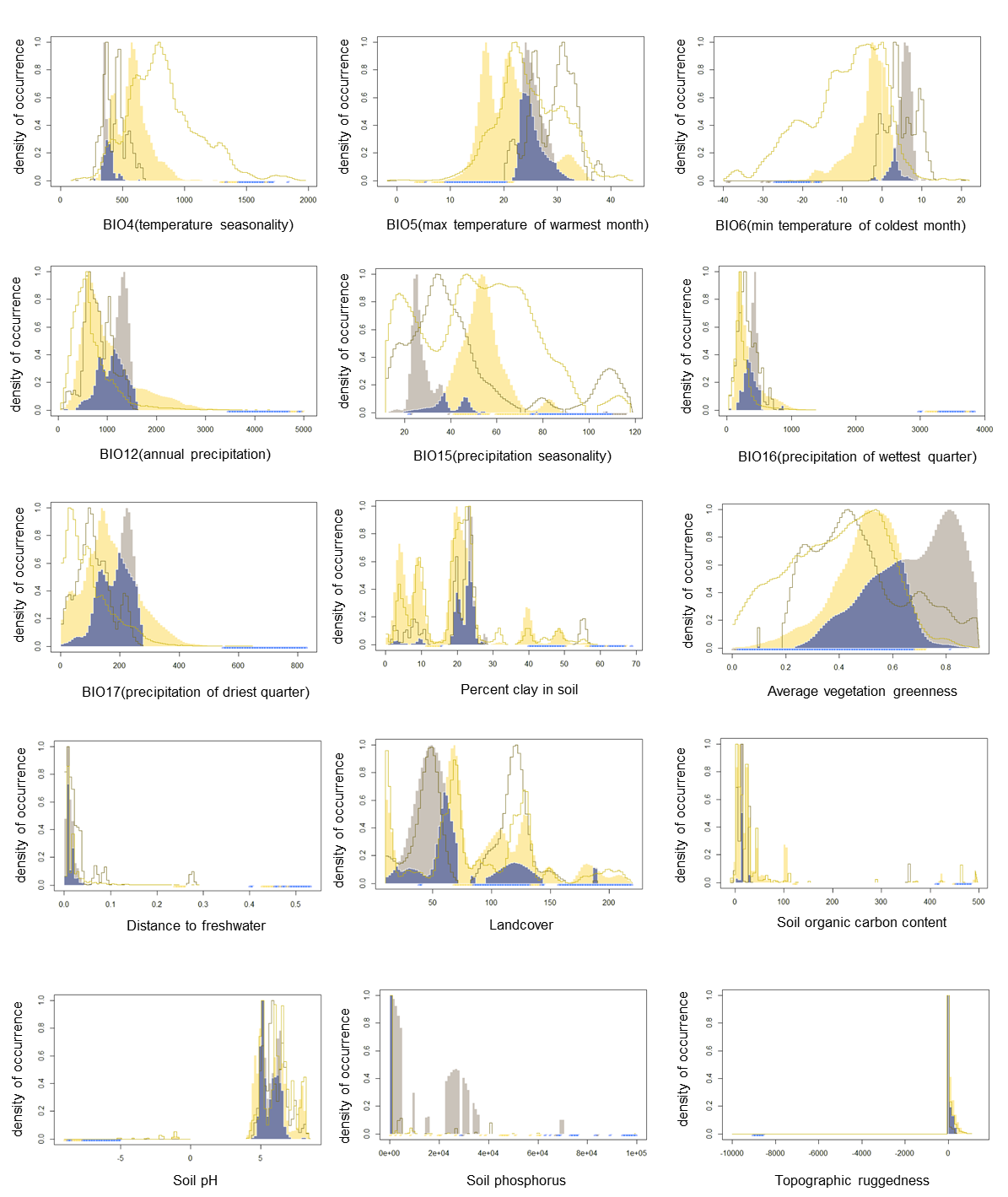


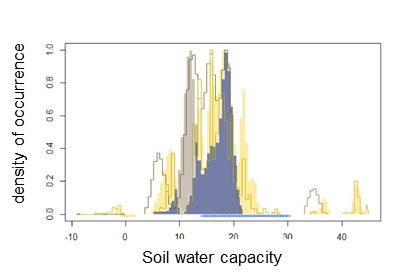


Figure 10. Red deer niche occupancy profiles for each variable. Tan represents the total range, and dark brown represents the Australian range. Purple represents the overlap between the two ranges. Overlapping peaks indicate similar variable tolerances. Tan solid lines represent 100% of available space in the total range (without Australia) and dark brown represents 100% of the available space in the Australian range. Descriptions of variables used, including units, are provided in Table 1.


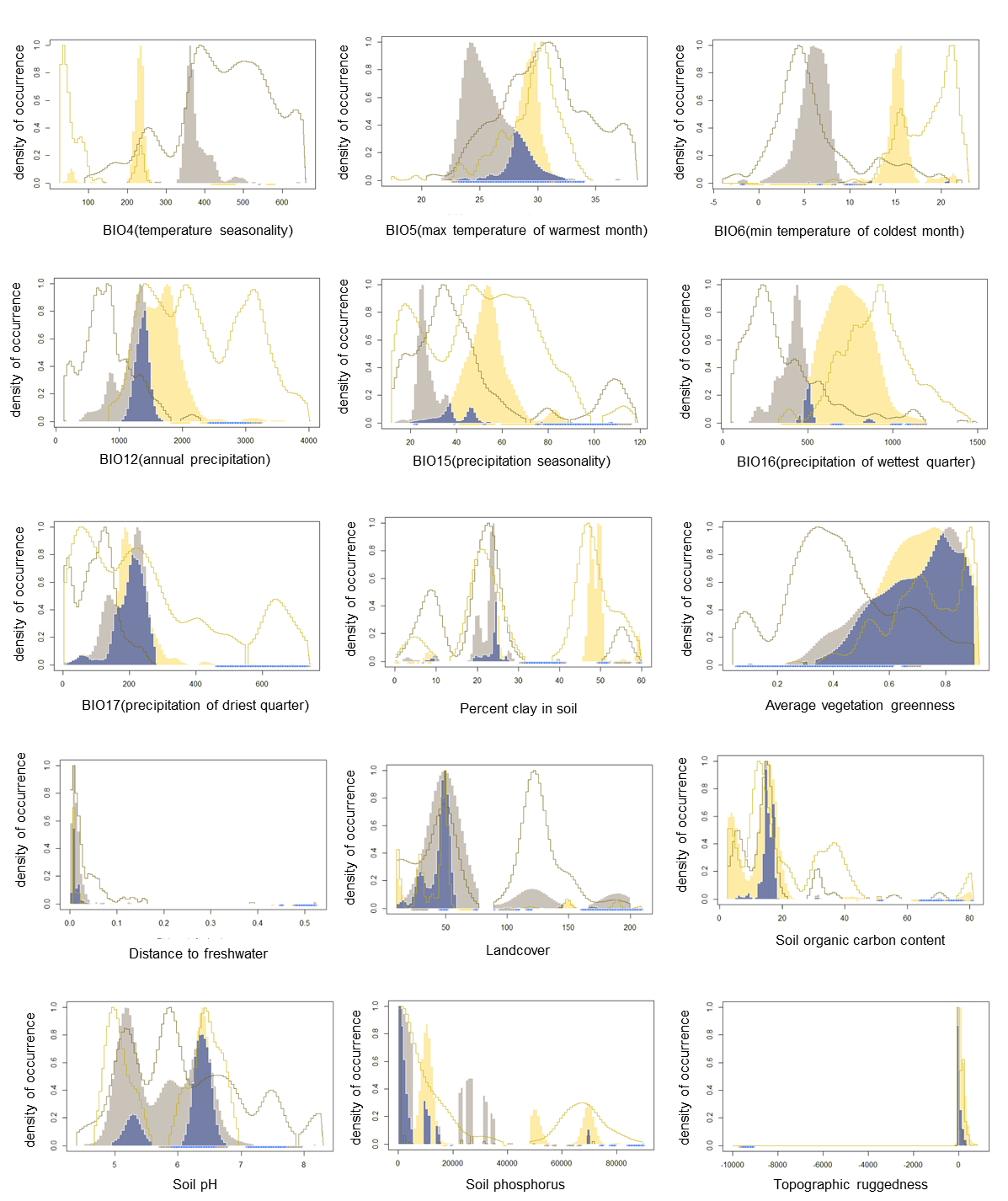


**
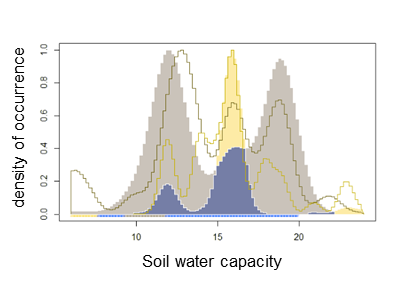
**

Figure 11. Rusa deer niche occupancy profiles for each variable. Tan represents the total range, and dark brown represents the Australian range. Purple represents the overlap between the two ranges. Overlapping peaks indicate similar variable tolerances. Tan solid lines represent 100% of available space in the total range (without Australia) and dark brown represents 100% of the available space in the Australian range. Descriptions of variables used, including units, are provided in Table 1.


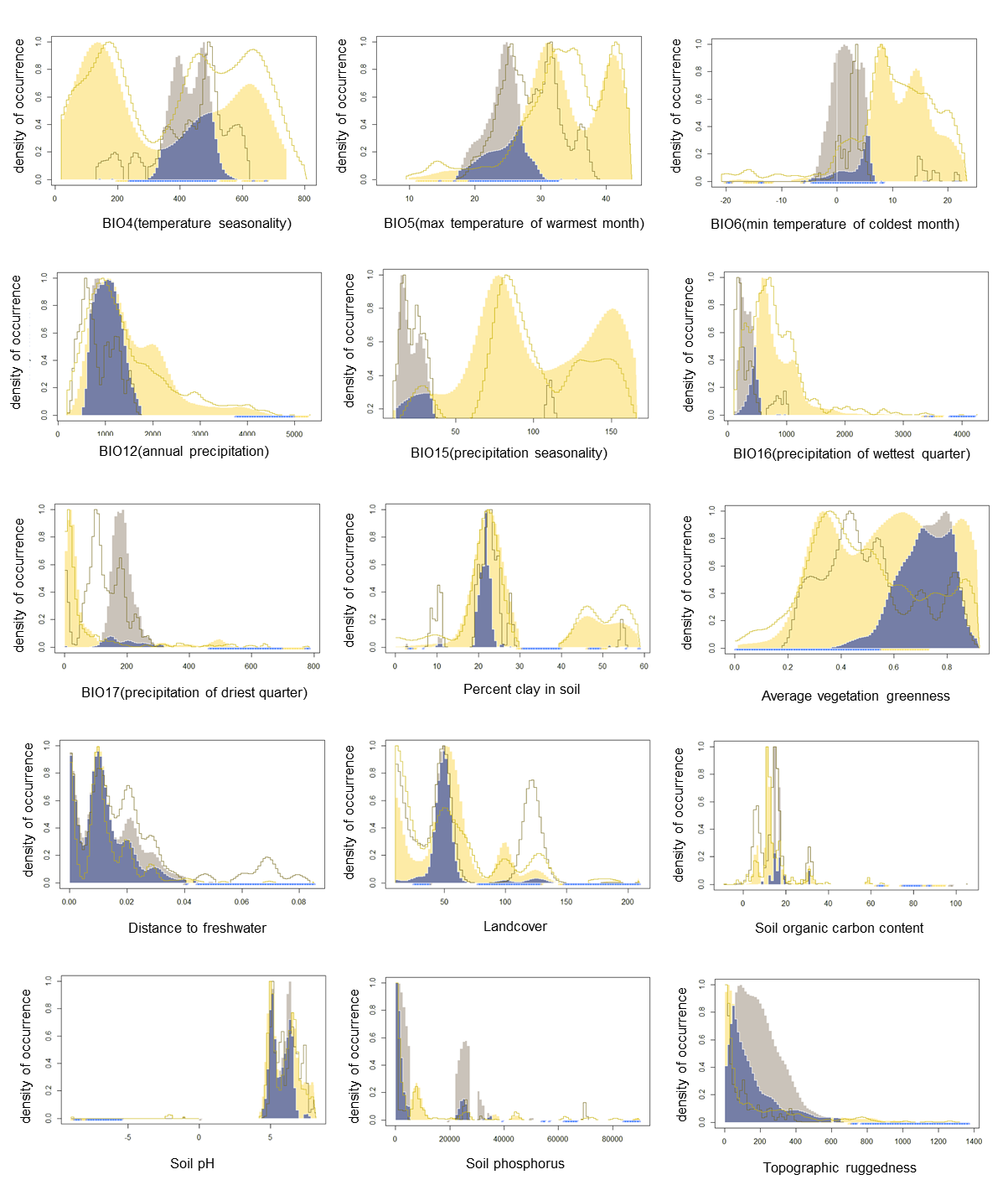


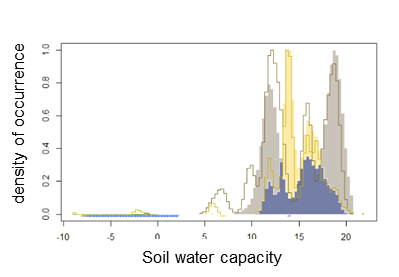


Figure 12. Sambar deer niche occupancy profiles for each variable. Tan represents the total range, and dark brown represents the Australian range. Purple represents the overlap between the two ranges. Overlapping peaks indicate similar variable tolerances. Tan solid lines represent 100% of available space in the total range (without Australia) and dark brown represents 100% of the available space in the Australian range. Descriptions of variables used, including units, are provided in Table 1.


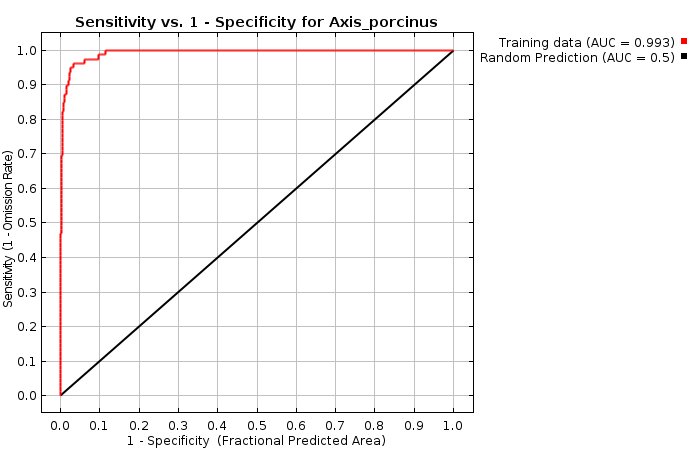

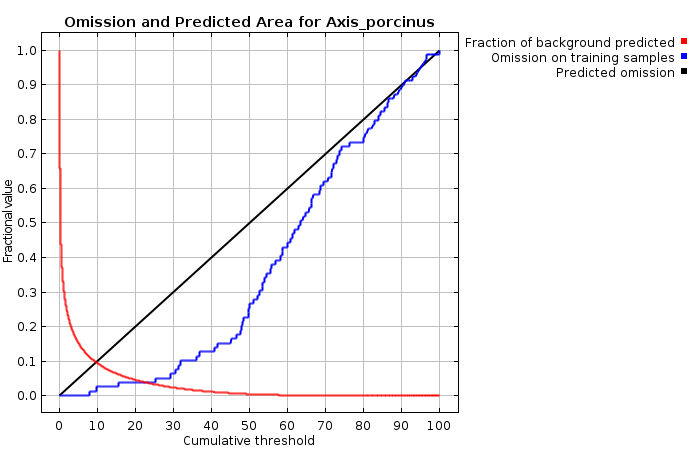

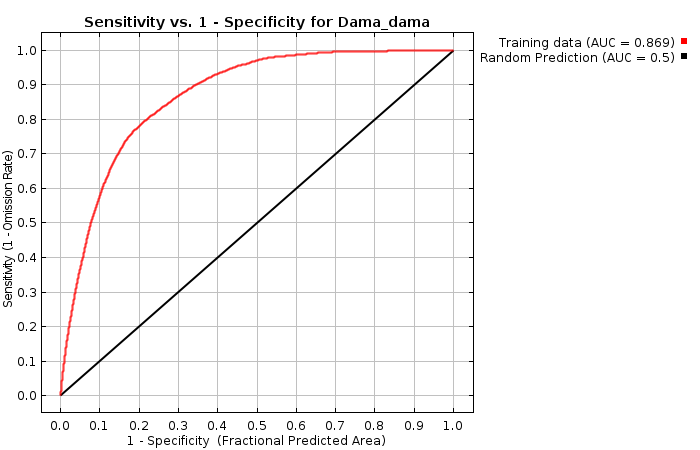

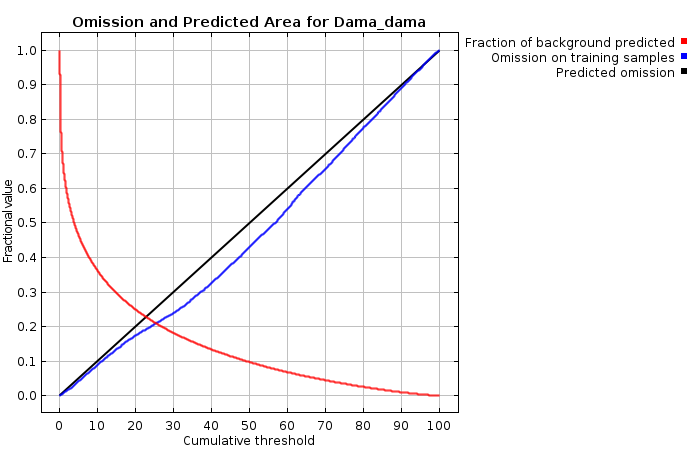

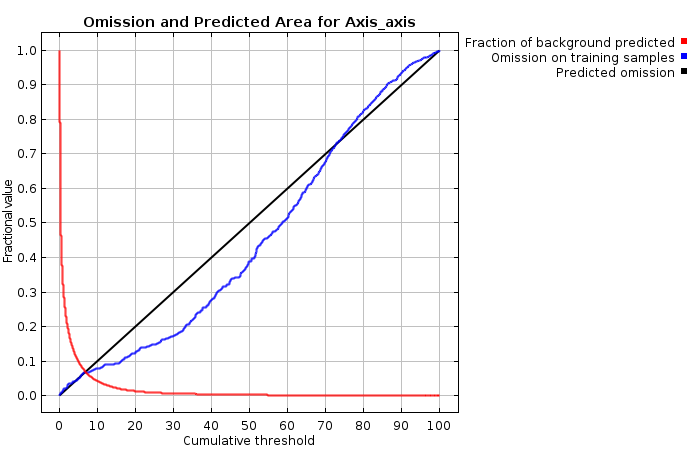

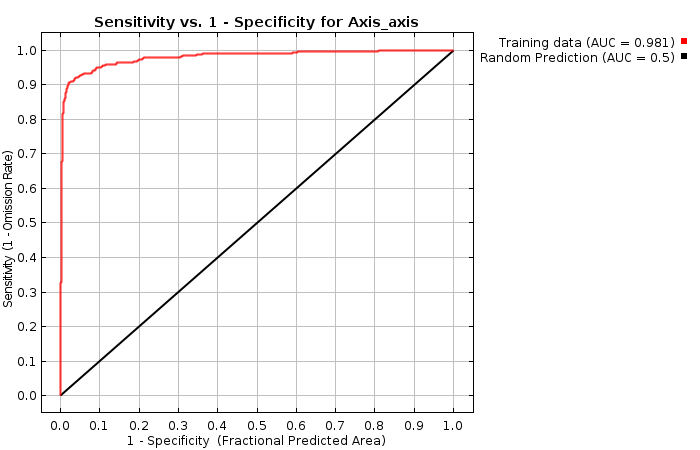


**c.**

**b.**

**a.**


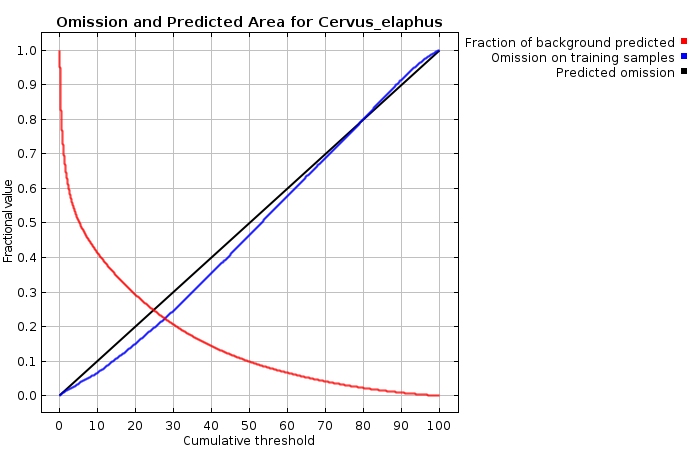

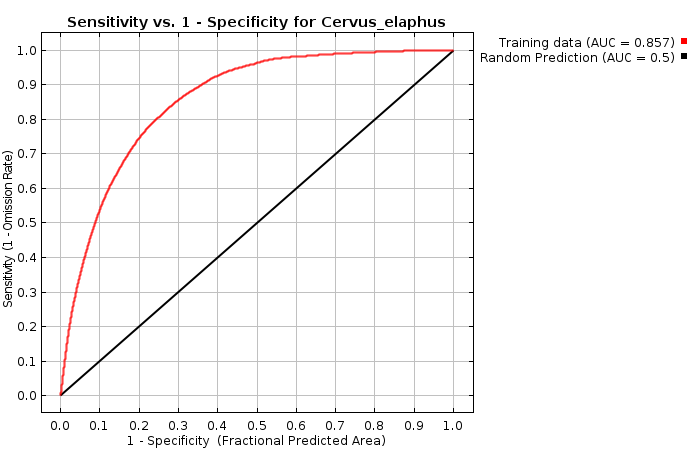


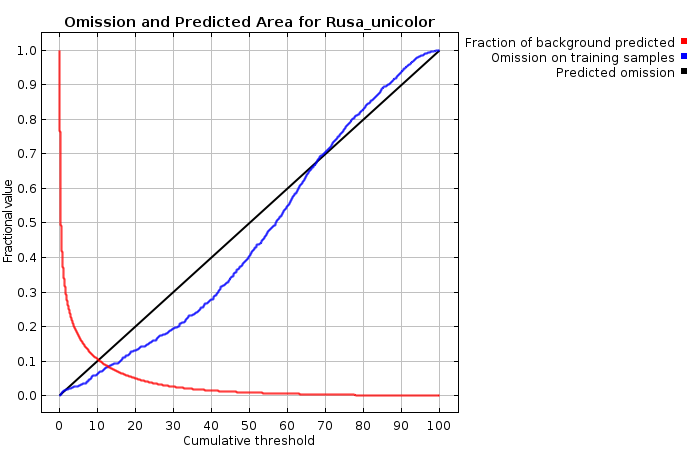

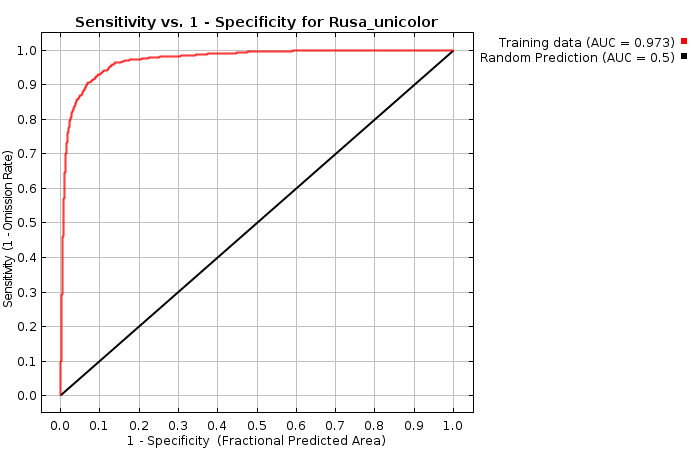

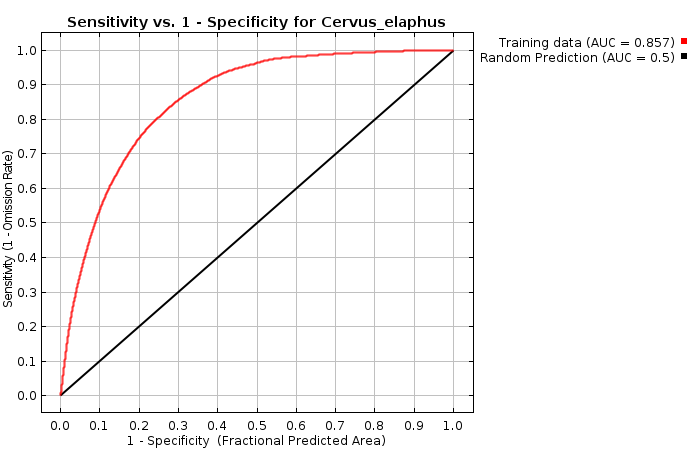

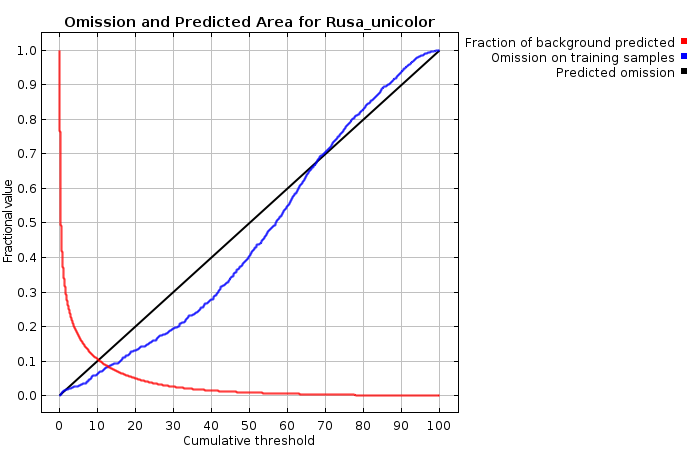

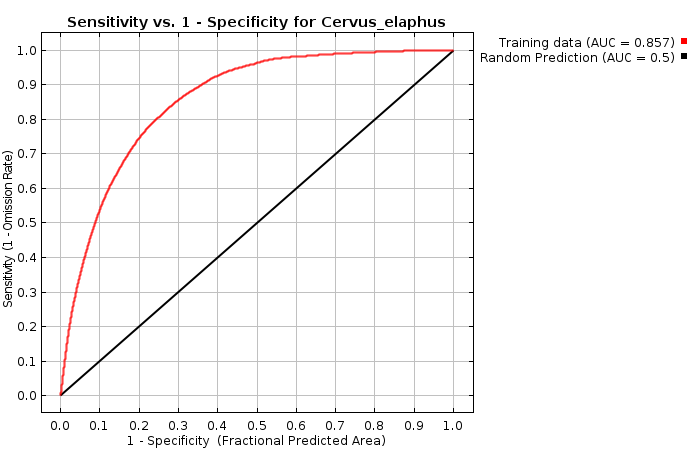

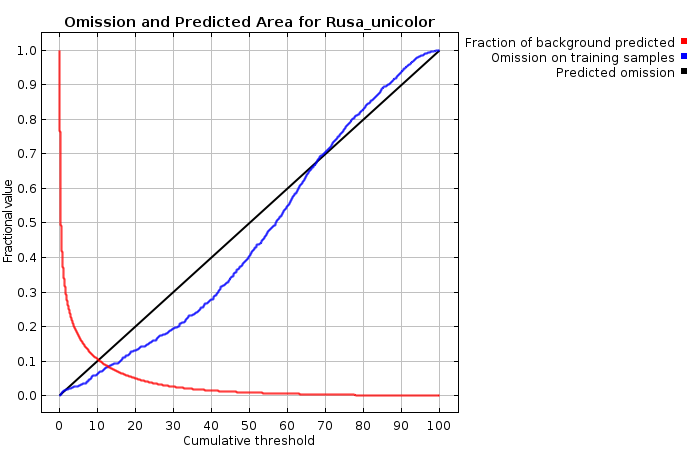

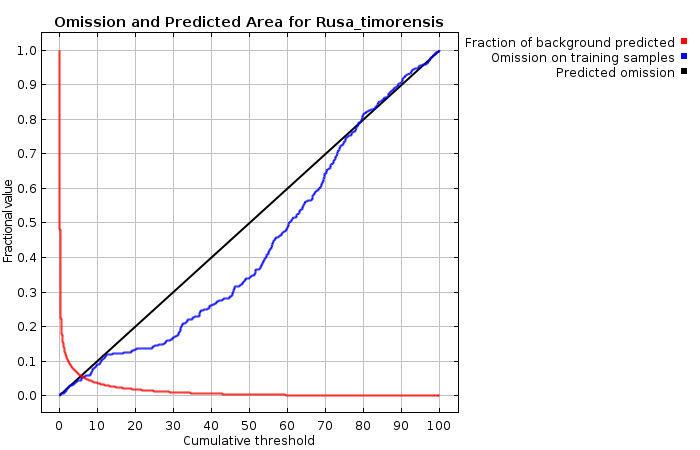

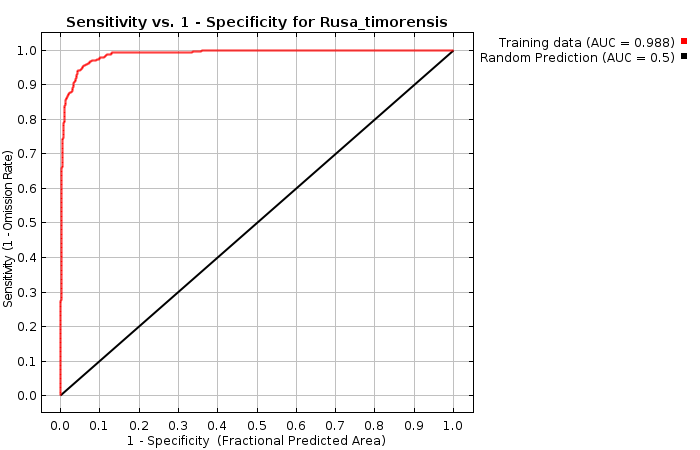

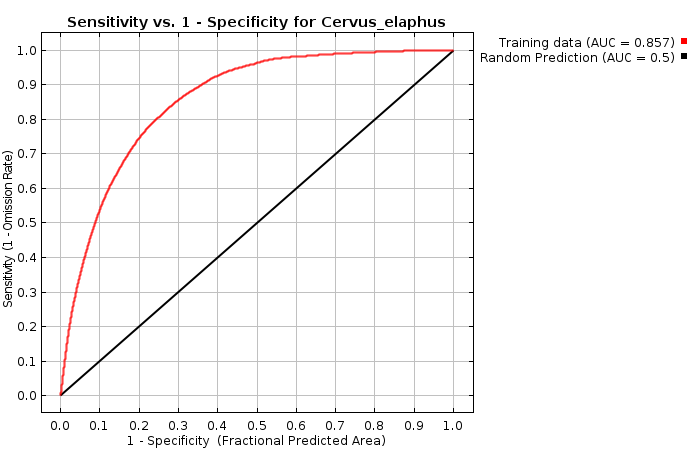

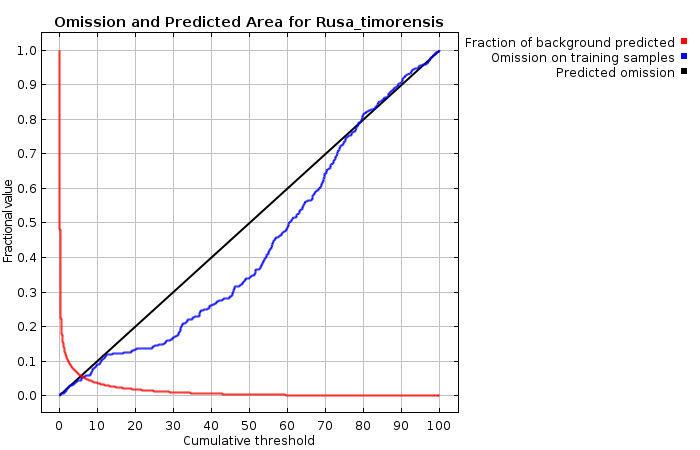

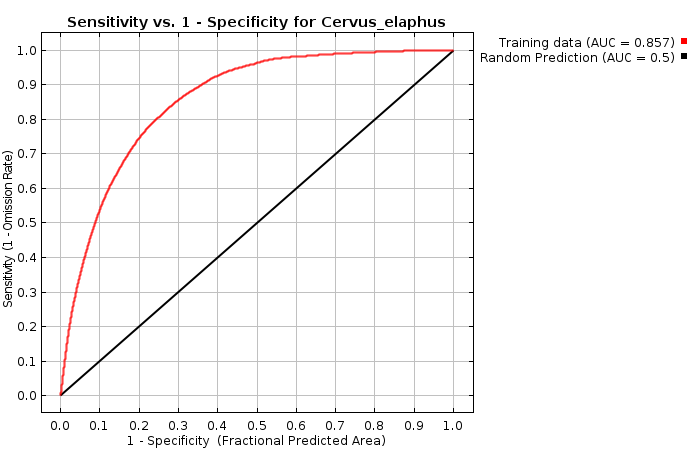

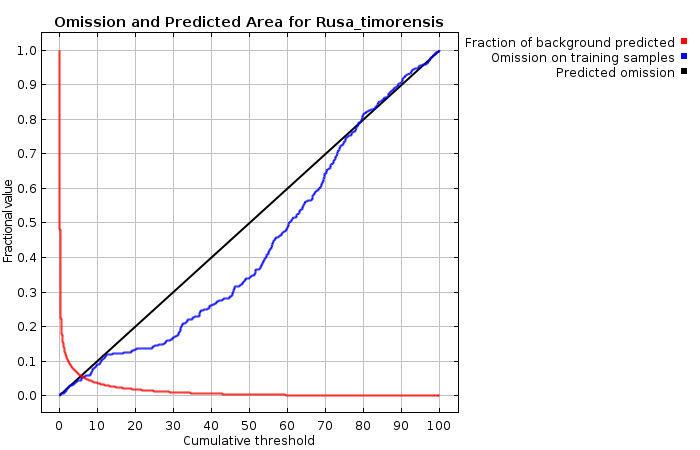


**f.**

**e.**

**d.**

Figure 13. Maxent receiver operating characteristic (ROC) curves for each of the six species of deer in Australia (a. chital, b. fallow, c. hog, d. red, e. rusa, f. sambar). On the left represents the omission rate and predicted area as a function of the cumulative threshold and on the right represents the ROC curve for the same data. The area under each ROC curve (AUC) is presented on the top right of each plot.


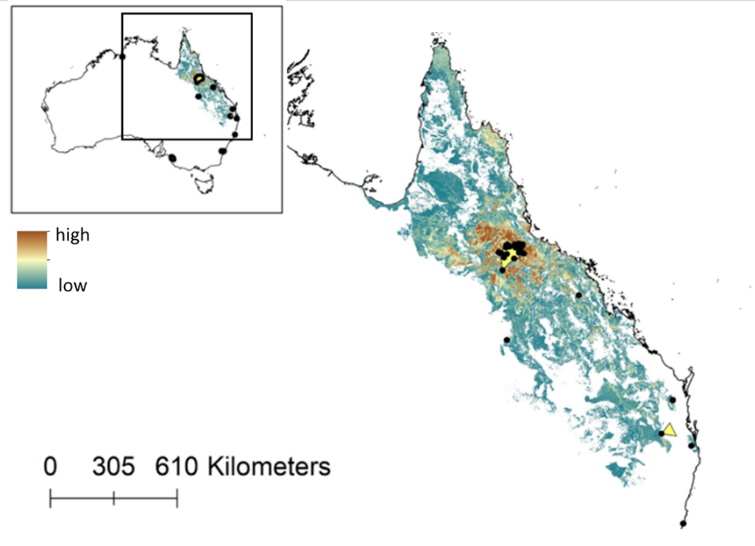

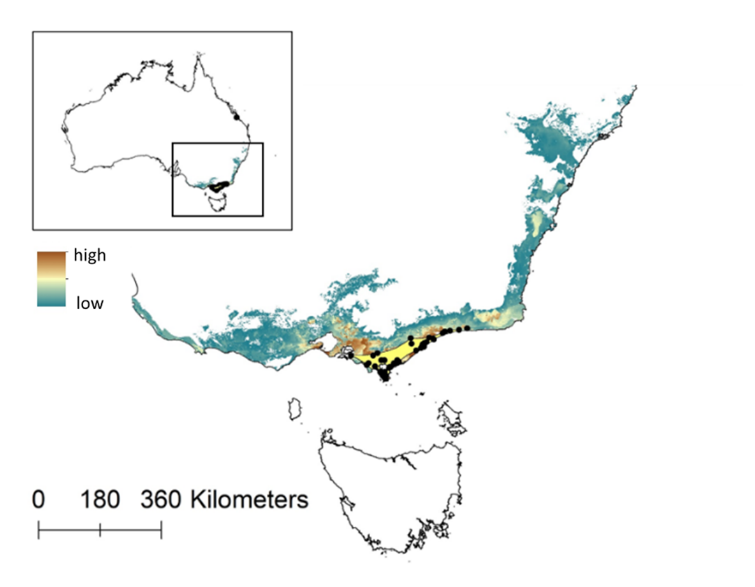


Chital deer

Hog deer


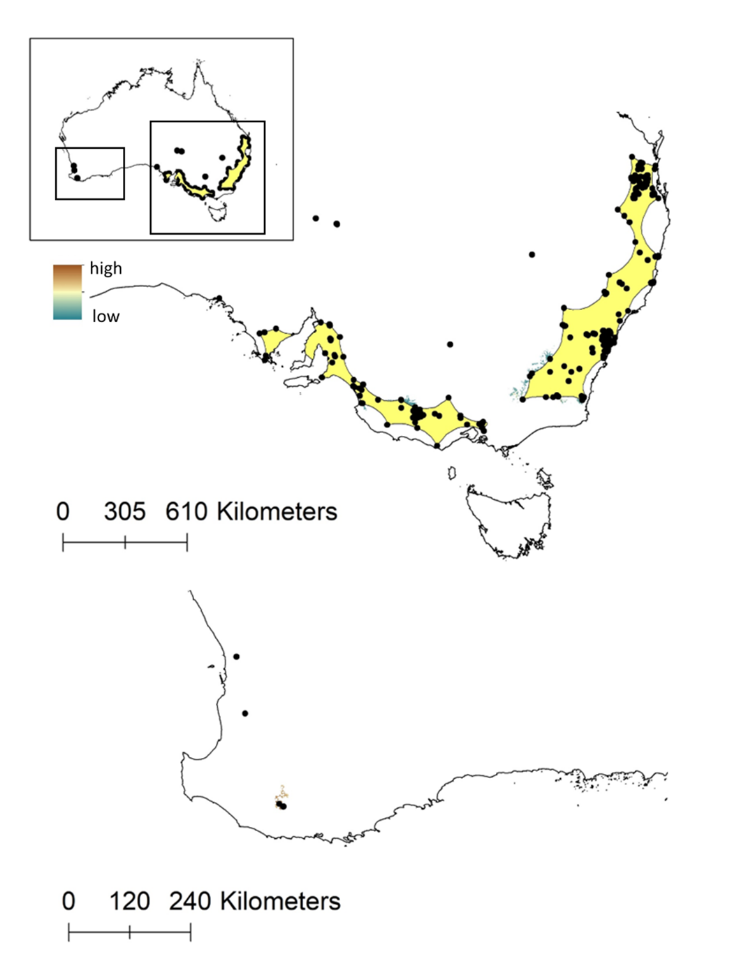

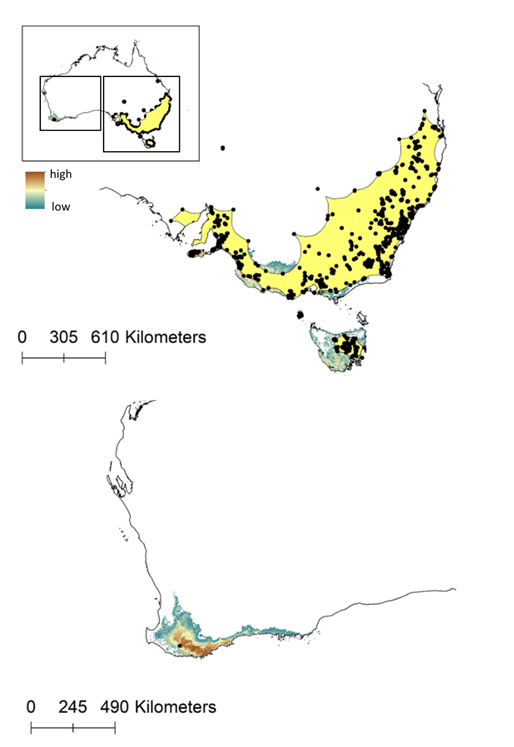


Red deer

Fallow deer


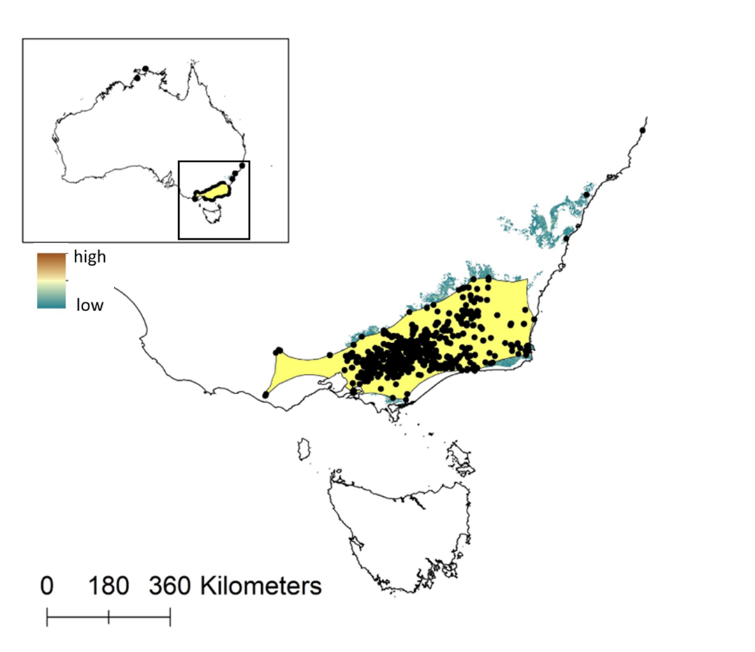

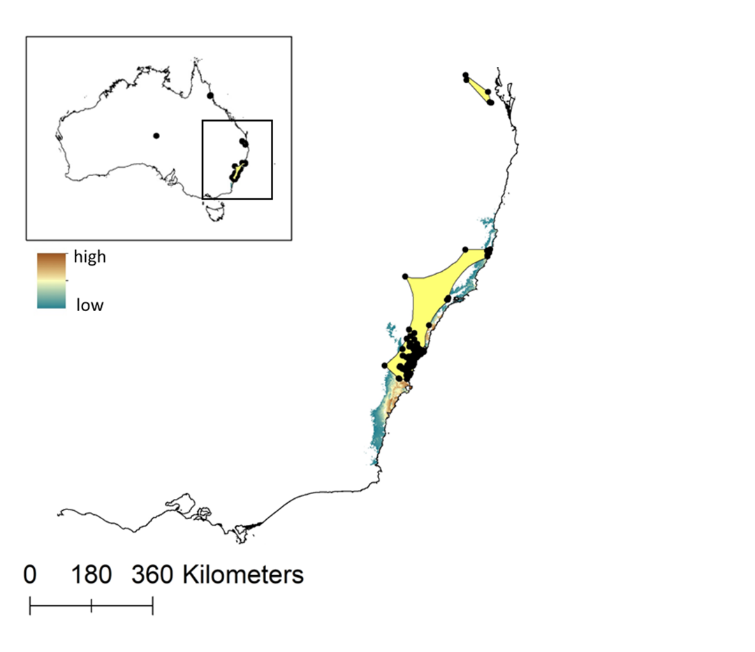


Rusa deer

Sambar deer

Figure 14. Maps of invasible habitat and future spread range from high vulnerability (brown) to low vulnerability (teal) as determined by MaxEnt modelling, including the present estimated ranges (yellow) and records (dots) of the six feral deer species in Australia.
